# Multiple selective sweeps of ancient polymorphisms in and around *LTα* located in the MHC class III region on chromosome 6

**DOI:** 10.1186/s12862-019-1516-y

**Published:** 2019-12-02

**Authors:** Michael C. Campbell, Bryan Ashong, Shaolei Teng, Jayla Harvey, Christopher N. Cross

**Affiliations:** 1 Department of Biology, College of Arts and Sciences, Howard University, Washington, DC 20059 USA; 2 Department of Anatomy, College of Medicine, Howard University, Washington, DC 20059 USA

**Keywords:** Archaic hominins, Human population genetics, MHC class III region, Balancing selection, Soft selective sweep

## Abstract

**Background:**

Lymphotoxin-α (*LTα*), located in the Major Histocompatibility Complex (MHC) class III region on chromosome 6, encodes a cytotoxic protein that mediates a variety of antiviral responses among other biological functions. Furthermore, several genotypes at this gene have been implicated in the onset of a number of complex diseases, including myocardial infarction, autoimmunity, and various types of cancer. However, little is known about levels of nucleotide variation and linkage disequilibrium (LD) in and near *LTα*, which could also influence phenotypic variance. To address this gap in knowledge, we examined sequence variation across ~ 10 kilobases (kbs), encompassing *LTα* and the upstream region, in 2039 individuals from the 1000 Genomes Project originating from 21 global populations.

**Results:**

Here, we observed striking patterns of diversity, including an excess of intermediate-frequency alleles, the maintenance of multiple common haplotypes and a deep coalescence time for variation (dating > 1.0 million years ago), in global populations. While these results are generally consistent with a model of balancing selection, we also uncovered a signature of positive selection in the form of long-range LD on chromosomes with derived alleles primarily in Eurasian populations. To reconcile these findings, which appear to support different models of selection, we argue that selective sweeps (particularly, soft sweeps) of multiple derived alleles in and/or near *LTα* occurred in non-Africans after their ancestors left Africa. Furthermore, these targets of selection were predicted to alter transcription factor binding site affinity and protein stability, suggesting they play a role in gene function. Additionally, our data also showed that a subset of these functional adaptive variants are present in archaic hominin genomes.

**Conclusions:**

Overall, this study identified candidate functional alleles in a biologically-relevant genomic region, and offers new insights into the evolutionary origins of these loci in modern human populations.

## Background

Lymphotoxin-α (*LTα*) in the MHC class III region encodes a potent cytotoxic polypeptide that plays a key role in regulating a number of biological processes, including lipid metabolism, coagulation, neurotransmission, and immunological response [1–12]. Studies have also shown that common variants in and/or near *LTα* contribute to the onset of complex diseases. For example, a recent analysis demonstrated that individuals with the A-allele at *rs*909253— located in an intronic region of *LTα*— have a higher risk for nasal NK/T-cell lymphoma relative to individuals with the G-allele in a Chinese population [13]. Data have also indicated that variability at *rs*2239704*, rs*909253, *rs*1041981, and *rs*2229094 was associated with elevated risk for other types of cancer, such as gastric and breast cancers in East Asians as well as non-Hodgkin lymphoma in individuals of European ancestry [14–20]. In vivo analyses using a mouse model further showed that upregulation of *LTα* in hepatocytic cells— infected by the hepatitis B or C virus — contributed to apoptosis and/or cell transformation leading to the development of hepatocellular carcinoma [21]. In addition, variability at *rs*909253, *rs*1800683 and *rs*1041981 has been correlated with increased susceptibility to cardiovascular disease in European, East Asian and/or Brazilian populations [10, 22–24]. Lastly, allelic variation at *rs*909253 and/or at *rs*2229094 has been implicated in the onset of inflammatory/autoimmune disorders, such as chronic periodontitis [25], ankylosing spondylitis [26], rheumatoid arthritis [27], systemic lupus erythematosus [28], vitiligo [29] and Sjogren’s syndrome [30] in individuals of non-African ancestry.

Despite the important role that *LTα* plays in complex traits, little is still known about levels of nucleotide variation and LD in and/or near this gene. Equally as important, the evolutionary processes that have shaped patterns of diversity in this region are similarly not known. These pieces of information are critical for identifying additional alleles in the *LTα* region that might contribute to phenotypes, including disease susceptibility. To address this gap in knowledge, we analyzed sequence variation across ~ 10 kbs on chromosome 6, encompassing the *LTα* gene and the 5′ region (and more broadly across the entire chromosome in some cases), in 2039 individuals from 21 distinct populations in the 1000 Genomes Project. Here, we observed striking patterns of variation in global populations, including an excess of intermediate-frequency alleles, the maintenance of multiple common haplotypes, and a deep coalescent time for variation (dating > 1 million years ago). We also identified a number of common alleles in or near *LTα* that are present in Neandertal and Denisovan genomes, further supporting the inferred ancient age of nucleotide variation. While these findings are generally consistent with a model of long-term balancing selection, we also observed extensive haplotype homozygosity on chromosomes carrying derived alleles primarily in non-African populations, suggestive of recent selection. Furthermore, a subset of these adaptive alleles were predicted to alter transcription factor binding site affinity and protein stability, suggesting they play a role in gene function. This latter finding could be highly informative for biomedical studies focused on the development of therapeutic interventions that mitigate, mimic or magnify the effects of these functional sites to combat diseases. Overall, our study identified candidate alleles that contribute to phenotypic variation, and offers additional insights into the evolutionary origins of these loci in modern human populations.

## Results

### Patterns of nucleotide variation

**Fig. 1:**
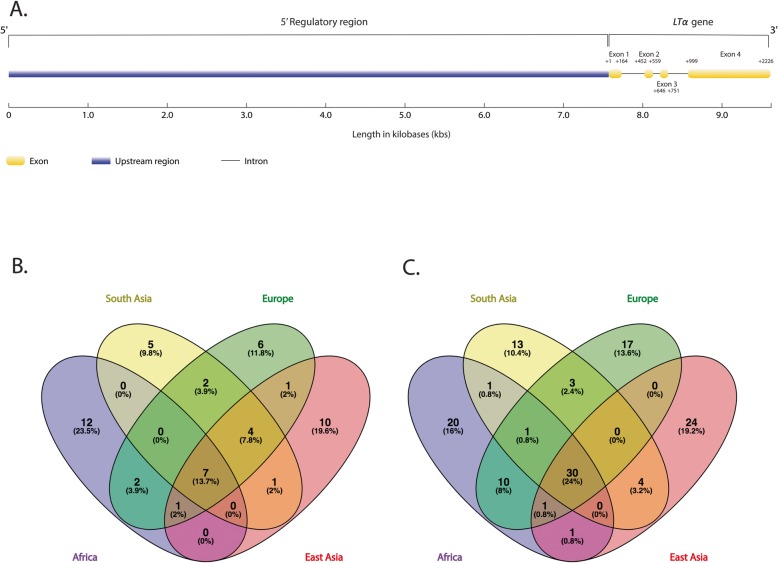
Gene structure and distribution of variants among major geographic regions. **a** Gene structure of *LTα* consists of four exons (yellow shapes) and intervening introns (black horizontal lines). Exons are labeled 1 through 4, and the start-end nucleotide positions for each exon are given in bps. The translation start site (ATG) begins at nucleotide position + 461 in exon 2; the entire *LTα* coding region is 618 bps in length. Immediately adjacent to the *LTα* gene is the 5′ regulatory region shown in purple. The combined length of *LTα* and the 5′ region is 9637 bps; **b** Venn diagram showing the number of polymorphisms in *LTα* found in four geographic regions— namely, Africa, South Asia, Europe and East Asia; **c** Venn diagram showing the number of polymorphisms in the 5′ regulatory region present in the above-mentioned geographic regions. For **b** and **c**, the number of polymorphisms shared among geographic regions is given in the interior portions of intersecting ovals, while the number of polymorphisms private to a particular geographic region is given outside of the intersecting ovals. We did not include African American and African Caribbean (Barbadian) populations with indigenous Africans when we examined the continental differences in the number of polymorphisms

We identified a total of 183 bi-allelic single nucleotide polymorphisms (SNPs) across ~ 10 kbs of sequence, encompassing *LTα* (2226 base pairs [bps]) and the adjacent 5′ non-coding region (7411 bps) on chromosome 6, in 21 global populations from the 1000 Genomes Project. Of the 183 polymorphisms, 52 SNPs were located within *LTα*, while the remaining 131 SNPs were found in the 5′ region (Additional file 2: Table S1). The *LTα* gene is comprised of four exons (Fig. 1); however, the mature LTα protein is encoded by exons 2 (from bps + 461 to + 559), 3 (from bps + 646 to + 751) and 4 (from bps + 999 to + 1411). In the *LTα* coding region, we identified 12 polymorphisms in global populations, eight of which were nonsynonymous changes. Two of these nonsynonymous polymorphisms (*rs*2229094 and *rs*1041981) occurred at relatively high frequency in all populations. More specifically, the minor allele frequency (MAF) at *rs*2229094 (C-allele) and *rs*1041981 (A-allele) ranged from 27.3 to 45.8% and from 20.2 to 33.8%, respectively, in South Asian populations. In Europeans, the *rs*2229094 minor C-allele and *rs*1041981 minor A-allele varied from 22.9 to 38.3% and from 24.8 to 30.9%, respectively, while the MAF at these loci ranged from 14.4 to 22.8% and from 38.4 to 54.8%, respectively, in East Asian populations. Likewise, the minor C-allele at *rs*2229094 and the minor A-allele at *rs*1041981 varied from 22.4 to 30.8% and from 36.3 to 61.5%, respectively, in populations of African descent (which include indigenous Africans, African Americans and African Caribbeans). Further comparative analysis also showed that allelic variation at *rs*2229094 was present in the genomes of two closely related archaic species to modern humans—Neanderthals and Denisovans (Additional file 2: Table S1).

In addition, we detected population-specific nonsynonymous variation in our modern human samples. For example, the minor alleles at *rs*538402044, *rs*562333039, *rs*538877791, *rs*566451995 occurred at < 2.5% frequency in non-Africans, while these alleles were absent in populations of African descent. Furthermore, the minor C-allele at *rs*2229092 was common in Europeans varying from 5.1 to 7.0%, while it occurred at lower frequency in South Asians (from 1.7 to 5.5%) and in East Asians (from < 1 to 2.4%). In contrast, the *rs*2229092 C-allele was absent in indigenous Africans, but was observed at relatively low frequency (~ 1–2.5%) in African American and African Caribbean populations. Additionally, we found that synonymous variants were either absent or occurred at very low frequency (< 1.0%) in all populations (Additional file 2: Table S1). Overall, we observed a striking deficit of synonymous SNPs relative to nonsynonymous polymorphisms in global populations.

In the intronic regions of *LTα*, we identified a total of 31 SNPs (Additional file 2: Table S1). Alleles at four of these polymorphic sites (*rs*1800683 *rs*909253, *rs*2239704, and *rs*746868) occurred at relatively high frequency in global populations (Additional file 2: Table S1). In particular, the MAF at *rs*1800683 and *rs*909253 both varied from ~ 20.2 to 33.8% frequency in South Asians, from 38.5 to 54.7% in East Asians, from 26.6 to 30.9% in Europeans, and from 38.9 to 64.1% in populations of African descent (Additional file 2: Table S1). Furthermore, the MAF at *rs*2239704 and *rs*746868 (Additional file 2: Table S1) ranged from ~ 31 to ~ 44.0% in South Asians, from 33.3 to 48.1% in Europeans, and from 24.1 to 47.1% in East Asians. Comparatively, the frequency of minor alleles at these sites was lower in African and African-descended populations, varying from ~ 16.0 to ~ 38.0%. Our analysis also uncovered a moderate level of population-specific variation; specifically, 25.8, 12.9, 9.7 and 22.6% of the intronic variants were private to populations of African, South Asian, European and East Asian descent, respectively (Additional file 2: Table S1; Additional file 1: Figure S1). Additionally, we found that human derived alleles at *rs*909253 and *rs*2239704 were shared with Neandertal and Denisovan hominins.

In the adjacent 5′ region, we identified a total of 131 SNPs, and uncovered extensive allele sharing among distinct populations (Additional file 2: Table S1). Notably, a subset of these polymorphisms (specifically, *rs*2009658, *rs*2844484 and *rs*915654) have previously been implicated in the onset of complex diseases [19, 31]. Like in the *LTα* gene*,* our analyses also showed that modern and archaic humans shared a number of derived alleles located in the upstream region (Additional file 2: Table S1).

Lastly, we calculated standard measures of nucleotide diversity (*θ*_π_ and *θ*_W_) in *LTα* and the upstream region, separately, for each global population (Table 1). We found that *θ*_π_ (the mean number of pairwise differences per nucleotide) in the *LTα *gene was similar across diverse populations. Specifically, *θ*_π_ ranged from 1.18 × 10^− 3^ to 1.39 × 10^− 3^ in populations of African descent; from 1.19 × 10^− 3^ to 1.33 × 10^− 3^ in South Asians, from 1.34 × 10^− 3^ to 1.48 × 10^− 3^ in Europeans and from 1.19 × 10^− 3^ to 1.33 × 10^− 3^ in East Asians (Table 1). Likewise, estimates of *θ*_W_ (nucleotide diversity calculated based on the number of segregating sites) did not vary greatly among populations (Table 1). In the 5′ region, we detected a similar pattern of nucleotide diversity among geographically distinct populations (Additional file 2: Table S2). Overall, we observed little difference in the level of nucleotide diversity between African and non-African populations across this ~ 10-kb region.

**Table 1:** Summary statistics based on sequence variation in the *LTα* gene

Population	2N	*S*	Singletons	*h*-diversity	θ_π_	θ_W_	*D*_*T*_	*H*	M-K test	D_N_	D_S_	P_N_	P_S_
ACB	192	16	3	0.721	1.34 × 10^−3^	1.25 × 10^− 3^	0.190	−0.247	0.400	0	2	3	1
ASW	120	12	1	0.760	1.39 × 10^−3^	1.02 × 10^− 3^	0.942	0.341	0.100	0	2	3	0
ESN	198	13	3	0.704	1.22 × 10^−3^	1.01 × 10^− 3^	0.511	−0.956	0.333	0	2	2	0
GWD	226	13	4	0.700	1.32 × 10^−3^	9.90 × 10^− 4^	0.816	0.204	0.333	0	2	2	0
LWK	198	10	2	0.660	1.18 × 10^−3^	7.80 × 10^− 4^	**1.208**	−0.439	0.333	0	2	0	2
MSL	162	10	1	0.709	1.29 × 10^−3^	8.00 × 10^− 4^	**1.449**	−0.007	0.100	0	2	3	0
YRI	214	15	5	0.742	1.36 × 10^−3^	1.15 × 10^− 3^	0.460	−0.107	0.333	0	2	2	0
BEB	168	13	2	0.741	1.19 × 10^−3^	1.04 × 10^− 3^	**0.364**	**1.340**	0.067	0	2	4	0
GIH	206	13	3	0.736	1.25 × 10^−3^	1.00 × 10^− 3^	**0.603**	**1.102**	0.100	0	2	3	0
ITU	198	13	1	0.763	1.27 × 10^−3^	1.01 × 10^− 3^	**0.638**	**0.896**	0.100	0	2	3	0
PJL	186	12	2	0.744	1.33 × 10^−3^	9.40 × 10^−4^	**1.014**	**0.681**	0.067	0	2	4	0
STU	198	14	4	0.734	1.33 × 10^−3^	1.09 × 10^− 3^	**0.570**	**0.569**	0.100	0	2	3	0
FIN	198	12	1	0.808	1.48 × 10^−3^	9.30 × 10^−4^	**1.433**	**1.365**	0.100	0	2	3	0
GBR	178	15	4	0.775	1.41 × 10^−3^	1.19 × 10^− 3^	**0.486**	**0.724**	0.100	0	2	3	0
IBS	214	15	3	0.728	1.34 × 10^−3^	1.15 × 10^− 3^	**0.416**	**0.285**	0.067	0	2	4	0
TSI	214	18	5	0.780	1.39 × 10^−3^	1.38 × 10^− 3^	0.011	**0.995**	0.400	0	2	3	1
CDX	186	8	2	0.616	1.19 × 10^−3^	6.30 × 10^−4^	**1.974**	−0.643	0.067	0	3	4	0
CHB	206	13	2	0.694	1.32 × 10^−3^	1.00 × 10^− 3^	**0.774**	0.138	0.143	0	2	4	1
CHS	210	9	3	0.610	1.19 × 10^−3^	6.90 × 10^−4^	**1.620**	−0.792	0.100	0	2	3	0
JPT	208	12	3	0.644	1.28 × 10^−3^	9.20 × 10^−4^	**0.942**	−0.440	0.429	0	2	3	2
KHV	198	15	5	0.687	1.33 × 10^−3^	1.17 × 10^− 3^	**0.369**	−0.009	**0.048**	0	2	5	0

Tajima’s D (*D*_T_) measures the difference between two estimates of nucleotide diversity, *θ*π and *θ*w. Fay and Wu’s H (*H*) measures an excess of high compared to intermediate frequency variants. Statistical significance for each statistic was determined by comparing observed estimates to expected values under different scenarios of population growth (Additional file 2: Tables S3 and S4) Numbers in bold indicate significance at *P* < 0.05. 2*N* is the number of gene copies analyzed in each population; *S* is the number of segregating sites; h is the number of haplotypes; and *h*-diversity is the haplotype diversity. The number of singletons is listed for each population. The number of silent polymorphic sites (P_S_), replacement polymorphic sites (P_N_), silent divergent sites (D_S_), and replacement divergent sites (D_N_) in the *LTα* coding region is also given. Statistical significance for the McDonald–Kreitman (M-K) test was determined using the Fisher’s exact test; significant (*P* < 0.05) values are given in bold

### Tests of neutrality

To determine if patterns of variation are consistent with neutral evolution, we calculated Tajima’s *D* (*D*_T_) and the Fay and Wu’s *H* (*H*) statistics for the *LTα* gene and the upstream region, separately, in each population (Table 1; Additional file 2: Table S2). We also generated expected *D*_T_ and *H* values under varying models of demographic growth using the ms software [32]. We incorporated growth as a parameter in these coalescent simulations given the genetic evidence for past human population expansion in prior studies [33, 34]. Based on these analyses, we found that *D*_T_ for *LTα* was more positive than expected (*P* < 0.05) in South Asians, Europeans and East Asians (Table 1; Additional file 2: Table S3). Moreover, we observed a significant departure of *H* values (*P* < 0.05) in South Asian and European populations (Table 1; Additional file 2: Table S4). Our results also showed a general pattern of positive *D*_T_ in populations of African descent, with the largest departures from expected values occurring in the Mende from Sierra Leone and the Luhya from Kenya (Table 1; Additional file 2: Table S3). However, we did not observe significant *H* values in African, African American and African Caribbean populations (Barbadians) (Table 1; Additional file 2: Table S4).

Additionally, we calculated *D*_T_ and *H* statistics for the adjacent 5′ region in each population (Additional file 2: Tables S5 and S6), and found significantly positive *D*_T_ values (*P* < 0.05) in South Asians, Europeans, and East Asians (Additional file 2: Tables S2 and S5). Furthermore, *H* statistics were more positive than expected (*P* < 0.05) in these populations (Additional file 2: Tables S2 and Table S6). In comparison, we observed a mix of positive and slightly negative *D*_T_ values (Additional file 2: Tables S2 and S5) among populations of African descent. However, we did detect significantly positive *H* values (*P* < 0.05) in both indigenous and recently admixed Africans (Additional file 2: Tables S2 and S6).

To further assess whether or not variation at *LTα* is evolving neutrally, we applied the McDonald-Kreitman (M-K) test, which compares the ratio of synonymous and non-synonymous sites within and between species, to our sequence data (Table 1). Our results showed a significant excess of nonsynonymous variants (*P* = 0.047) in the Kinh population from Vietnam and a borderline significant excess of amino acid variation (0.1 ≥ *P* ≥ 0.05) in other populations of African (African Americans and the Mende), South Asian (Bengali, Gujarati, Telugu, Punjabi and Sri Lankan Tamil), European (Finnish, Great British, and Iberian), and East Asian (Dai and southern Han Chinese) ancestry (Table 1). Thus, we consistently observed a higher proportion of nonsynonymous polymorphisms relative to synonymous changes in all populations. Overall, the excess of nonsynonymous variants and the sharply positive *D*_T_ and *H* statistics in global populations are consistent with the action of long-term balancing selection [35–37].

### Haplotype variation and inferred relationships

We analyzed phased haplotype data, encompassing the entire 9637 base pair (bp) region, and identified 223 distinct haplotypes in global populations (Additional file 2: Table S7A). Of these variants, 23 haplotypes (H6, H8, H12, H30, H36, H37, H80, H82, H86, H105, H119, H128, H132, H136, H142, H158, H160, H187, H190, H204, H214, H217 and H219) were common, and six of these haplotypes were found in all populations (Additional file 2: Table S7A); 16–18 common haplotypes accounted for 80.4–88.4%, 77.6–83.1% and 77.1–88.9% of the total number of haplotypes in South Asian, European and East Asian populations, respectively (Additional file 2: Table S8). Comparatively, a smaller number of common haplotypes (H6, H30, H37, H82, H86, H119, H128, H142, H187, H190, H217 and H219) comprised 66.7–74.2% of all haplotype lineages in African and African-descended populations (Additional file 2: Table S9). Further analysis of all haplotype variation also showed that Africans and non-Africans exhibited high levels of haplotype diversity (*h*-diversity). Specifically, *h*-diversity varied from 90.5 to 95.0% in populations of African descent; from 91.5 to 93.6% in South Asians; from 91.9 to 93.3% in Europeans; and from 85.9 to 89.9% in East Asians (Table 1).

**Fig. 2:**
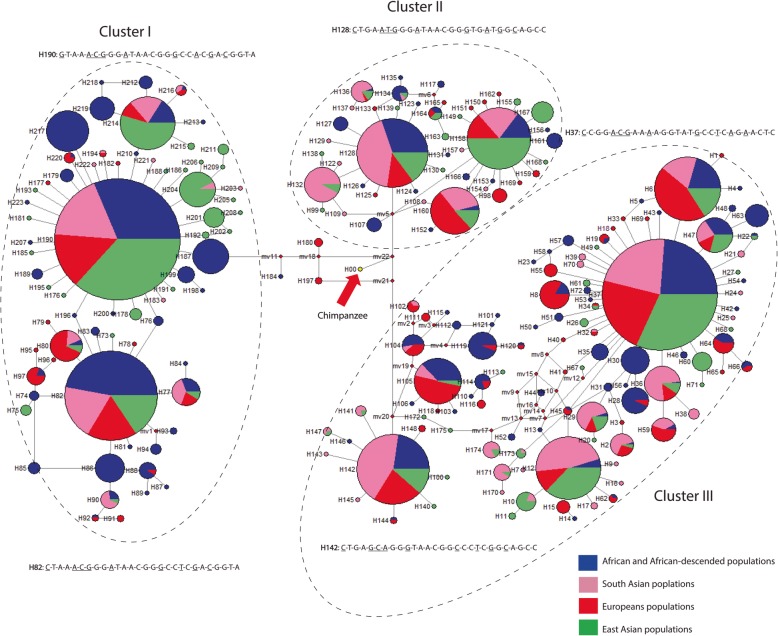
Haplotype network of global variation across ~ 10 kbs encompassing *LTα* and the 5′ region. Haplotype relationships were constructed using the median-joining algorithm implemented in Network 5.0 [38]. The resulting phylogeny consists of nodes (representing haplotypes), and links which connect the nodes in the shortest possible tree. The size of the nodes is proportional to the number of chromosomes with a given haplotype, and the colors within haplotypes (or nodes) indicate the frequency of that haplotype in pooled populations (such as, South Asians or East Asians). Distinct clusters of haplotypes are surrounded by circles/ovals with dashed lines, and the alleles that define the highest-frequency haplotype(s) are also given in each cluster. Underlined nucleotides indicate alleles that are inferred to be under selection and are shared with archaic hominins. Each haplotype cluster was arbitrarily labelled as Cluster I, Cluster II and Cluster III

We also performed a median-joining network analysis [38] to explore the phylogenetic relationships among haplotype lineages, and found that haplotype variation grouped into three distinct clusters (arbitrarily labeled I, II, III; Fig. 2). Each cluster consisted of low-frequency haplotypes radiating from common haplotypes in a “star-like” pattern which is the genetic pattern predicted under a model of positive or purifying selection [39]. Moreover, the highest-frequency haplotypes (specifically, H37, H82, H128, H142 and H190) in clusters I, II and III were shared among globally diverse populations, and these haplotype lineages differed from one another at 28 polymorphic sites. Intriguingly, we also found that 22 of the 28 polymorphic sites on these high-frequency haplotypes were present in the Neandertal and Denisovan genomes (Additional file 2: Table S7B).

### Pairwise linkage disequilibrium in the *LTα* region and surrounding genes

**Fig. 3:**
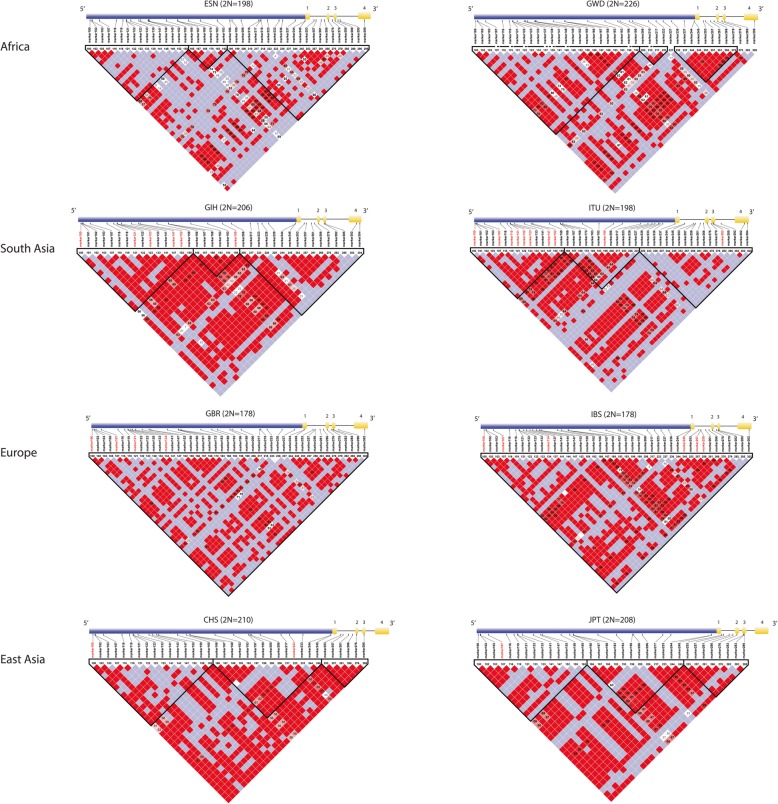
Pairwise LD across ~ 10 kbs encompassing *LTα* and the 5′ region in selected populations. We measured pairwise LD, using the *D*′ statistic, calculated with Haploview [40], which outputs a graphical representation of allelic associations. In the resulting plots, pairwise LD coefficients *D*′ × 100 are given in each cell. The color scheme of each cell signifies the strength of association between SNP alleles. Specifically, bright red squares indicate statistically significant LD between SNP pairs (*D*′ = 100; logarithm of odds (LOD) > 2), while shades of pink/white cells signify little evidence of LD (*D*′ < 100; LOD < 2) between loci and purple cells signal high LD but with little statistical support (low LOD) [40]. The bold triangles in the plots also indicate strong blocks of LD between SNP markers. The corresponding genomic coordinate for each marker can be found in Additional file 2: Table S10. The genomic region analyzed is given at the top of each plot (exons are labeled 1 through 4) The genomic region analyzed is given at the top of each plot (exons are labeled 1 through 4)

To quantify the allelic associations between SNPs across ~ 10 kbs, encompassing *LTα* and the 5′ region, we estimated pairwise LD using the *D′* statistic [40]. Our results generally showed a faster decay of LD between loci (*D*′ < 100) in populations of African ancestry compared to non-Africans. For example, in the Esan from Nigeria, we observed lower levels of allelic association (*D*′ < 100) within and between *LTα* and the upstream region (Fig. 3; Additional file 1: Figure S1; Additional file 2: Table S10), implying a history of recombination in this population*.* By contrast, our LD plots revealed a higher level of allelic association in South Asians, Europeans, and East Asians. In particular, we observed a greater number of instances of complete LD (D′ = 100) between loci in non-Africans compared to populations of African descent (Fig. 3; Additional file 1: Figure S1; Additional file 2: Table S10). The inference of recombination across *LTα* and the 5′ region is further supported by our phylogenetic network which displayed a small number of reticulations or loops among haplotype lineages (Fig. 2). It is known that these reticulate (non-bifurcating) relationships among haplotypes can arise due to historical recombination events [41].

To explore possible gene-gene interactions, we also quantified pairwise LD between genetic markers in the *LTα* region and neighboring genes (specifically, *NFKBIL1*, *TNFα*, and *LTβ*) within a 35,538-bp region on chromosome 6 (Additional file 1: Figure S2). This analysis showed that African and African-descended populations exhibited less LD (*D*′ < 100) between SNPs in the *LTα* region and nearby genes (Additional file 1: Figure S2). In comparison, however, we observed more instances of complete LD (*D*′ = 100) across this ~ 35.5 kilobase (kb) region in Eurasian populations (Additional file 1: Figure S2).

### Long-range haplotype structure

**Fig. 4:**
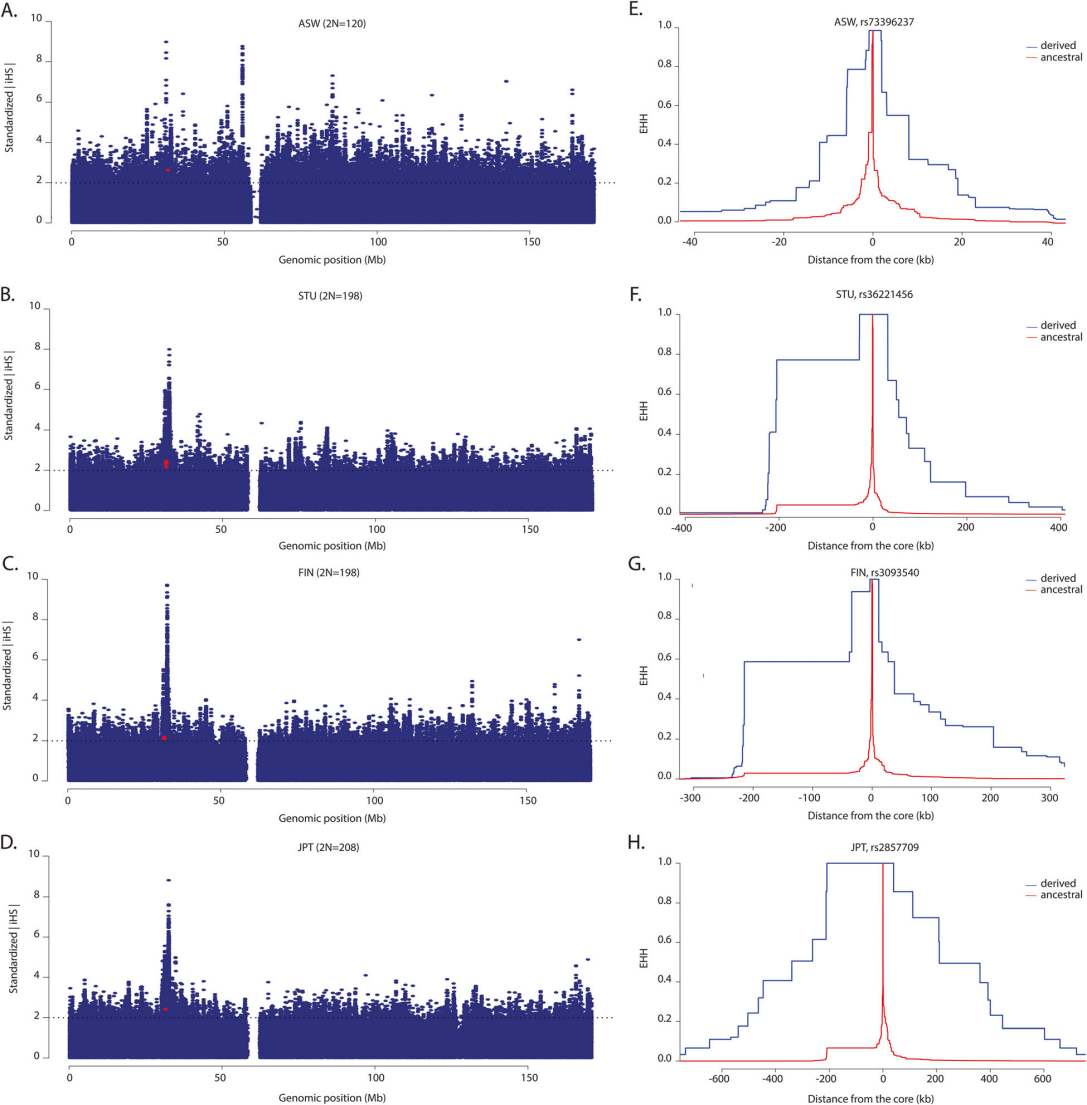
Integrated haplotype score (*i*HS) and extended haplotype homozygosity (EHH) plots for selected populations. **a**–**d** Manhattan plots of standardized |*i*HS| statistics for selected populations. The dashed horizontal lines represent the cut-off for extreme |*i*HS| scores (|*i*HS | > 2), representing the most extreme 5% of empirical |*i*HS| values. The red dots in plots represent outlier SNPs in and/or near *LTα*; (E–H) EHH plots for selected populations show the decay of identity of haplotypes on chromosomes with a derived allele (the blue line) versus an ancestral allele (the red line) at core SNPs as a function of distance. The distance from the core SNP (at zero) is displayed on the x-axis; the negative numbers indicate distance upstream from the core SNP, while positive values indicate distance downstream from the core SNP. The EHH values are shown on the y-axis. The distance from the core SNP (at zero) is displayed on the *x*-axis and the EHH values are shown on the *y*-axis. It is important to note that these EHH plots are selected examples from a larger set of EHH curves that were generated in the present study, which can be found in Additional file 1: Figure S5)

We further characterized long-range LD across the entire ∼170 megabase (Mb) region of chromosome 6 for each population using the *i*HS statistic [42]. In particular, we calculated the absolute standardized |*i*HS| scores by normalizing raw values with the selscan and norm software [43]. Based on this analysis, we observed outlier |*i*HS| statistics for multiple SNPs— namely, *rs*4947326, *rs*4947327, *rs*9267497, *rs*36221456, *rs*73396237, *rs*148407582, *rs*9267499, *rs*2857709, *rs*2857708 and *rs*2229092— in the coding and 5′ regions in South Asians (Fig. 4; Additional file 1: Figure S3; Additional file 2: Table S12). We also identified multiple SNPs with elevated |*i*HS| values in the upstream, intronic and exonic regions in European populations (Fig. 4; Additional file 1: Figure S3; Additional file 2: Table S12). In addition, we detected several outlier |*i*HS| scores for loci in the 5′ region only (*rs*73396237, *rs*915654, *rs*2857709) in populations from East Asia (Fig. 4; Additional file 1: Figure S3; Additional file 2: Table S12). We did not, however, observe outlier values for SNPs in the *LTα* gene or the 5′ region in populations of African descent, except in African Americans where we detected a single extreme |*i*HS| score for *rs*73396237 located in the upstream region (Fig. 4; Additional file 1: Figure S3; Additional file 2: Table S12).

To complement our |*i*HS| scans, we applied the *n*SL statistic which is a test for detecting both soft and hard sweeps in genomic data [44]. One advantage of this statistic is that it does not depend on the recombination rate, which can lead to false positive signals of haplotype homozygosity in chromosomal regions with low levels of recombination [44]. Using this method, we observed extreme standardized |*n*SL| scores (defined as |*n*SL| > 2) for variation in the 5′ region (*rs*36221456, *rs*73396237, *rs*2857709, *rs*4947326, *rs*2857601, *rs*2857708) and in the *LTα* coding region (*rs*2229092) in South Asians (Additional file 1: Figure S4; Additional file 2: Table S11), generally overlapping with the |*i*HS| results. We also detected outlier |*n*SL| values for a similar set of loci upstream (*rs*73396237, *rs*2857709, *rs*3093540, *rs*2071590, *rs*4947326, *rs*2857601, *rs*2239704 and *rs*3093546) and within the *LTα* gene (*rs*3093542 and *rs*2229092) in European populations (Additional file 1: Figure S4; Additional file 2: Table S12). Furthermore, we found a single extreme |*n*SL| score for *rs*73396237 in the upstream region in East Asians. By contrast, we did not observe unusual |*n*SL| scores for loci in any population of African descent (Additional file 1: Figure S4; Additional file 2: Table S12).

We also plotted the decay of haplotype homozygosity from core SNPs (represented by loci with extreme |*i*HS| or |*n*SL| scores) using the EHH statistic [45]. This analysis revealed long-distance haplotype homozygosity, spanning up to 1.4 megabases (Mbs), on chromosomes carrying the derived allele relative to chromosomes with the ancestral allele mainly in Eurasian populations (Fig. 4; Additional file 1: Figure S5). For example, we detected unusually long haplotype structure around derived alleles at *rs*73396237, *rs*2857709, *rs*2857708, *rs*4947326, *rs*4947327, *rs*148407582, *rs*9267497, *rs*9267499, and *rs*2229092 in the South Asian Telugu (ITU) population (Fig. 4; Additional file 1: Figure S5). We also observed a similar pattern of EHH on chromosomes carrying derived alleles in other non-African populations. In addition, we detected long-range EHH around the derived G-allele at *rs*73396237 (extending no more than 80 kbs) in African Americans in agreement with our |*i*HS| results (Fig. 4; Additional file 1: Figure S5). We did not, however, observe a difference in EHH curves for chromosomes harboring the derived versus the ancestral allele in other populations of African descent (Additional file 1: Figure S5).

### Population differentiation and structure

To examine patterns of genetic differentiation, we calculated mean *F*_ST_ among geographic regions (namely Africa, South Asia, East Asia, and Europe) using polymorphisms across the ~ 10 kbs encompassing *LTα* and the upstream region. Mean global *F*_ST_ was estimated to be 0.010132, much lower than *F*_ST_ estimates for autosomal loci, which typically range from 0.10 to 0.16 [46, 47]. Our analyses also revealed that per-site *F*_ST_ values for common SNPs were below 0.10 (Additional file 2: Table S1). For example, *F*_ST_ values for *rs*1800683, *rs*2239704, *rs*909253, *rs*746868, *rs*2229094, *rs*3093542, *rs*2229092 and *rs*1041981 within the *LTα* gene were estimated to be 0.069033, 0.020427, 0.068599, 0.021288, 0.029649, 0.026510, 0.034892 and 0.067877, respectively (Additional file 2: Table S1). Similarly, *F*_ST_ values for common SNPs in the 5′ region varied from 0.0055 to 0.0745 (Additional file 2: Table S1). Although the above estimates of *F*_ST_ were not outliers in the empirical distribution of *F*_ST_, calculated for ~ 7.5 million genome-wide SNPs, they did fall within the 15th percentile of empirical values. Thus, we consistently observed low estimates of *F*_ST_ in both the *LTα* gene and the upstream region.

### Inferred ages of variants in *LTα* and the adjacent 5′ region

Using a coalescent-based method [48], we inferred the mean time to the most recent common ancestor (TMRCA) for variation in both *LTα* and the upstream region, and calculated the mean ages of individual polymorphisms (in years ago ± standard deviation in years) (Additional file 2: Table S13). Our analysis revealed a deep coalescence time for the origin of variation, estimated to be ~ 1.0 million years ago (ya) ± 57,207.68 years. We also found that the ages of common polymorphisms in the 5′ region ranged from 200,571.46 ya (± 77,877.56 years) to 734,694 ya (± 140,326.55 years) (Additional file 2: Table S13). Furthermore, variants with the oldest inferred ages—namely, *rs*2857710 (734,694 ya ± 140,326.55 years), *rs*3131641 (453,306.20 ya ± 69,869.40 years), *rs*2857602 (718,530.73 ya ± 141,061.25 years), *rs*2844486 (626,693.98 ya ± 177,795.95 years) and *rs*1121800 (472,408.24 ya ± 106,530.63 years)—were also found in the Neanderthal and/or Denisovan genomes (Additional file 2: Tables S1 and S13). Additionally, several of the SNPs with deep coalescence times (i.e., *rs*4947326, *rs*4947327, *rs*148407582 and rs9267497) were inferred to be targets of recent selection based on our long-range haplotype analyses (Additional file 2: Tables S11 and S13).

### Inferred functional consequences

Given the higher proportion of nonsynonymous polymorphisms relative to synonymous changes in the *LTα* coding region, we inferred the possible effects of nonsynonymous SNPs on protein function using SIFT [49]. SIFT classifies nonsynonymous substitutions as either “tolerated” or “deleterious” based on sequence homology and the properties of amino acids [49]. This method predicted that seven of the eight nonsynonymous polymorphisms (specifically, *rs*538402044, *rs*2229094, *rs*368539892, *rs*2229092, *rs*1041981, *rs*538877791 and *rs*566451995) were “tolerated”, while variation at *rs*562333039 was predicted to be “deleterious”. Moreover, three “tolerated” variants— *rs*2229094, *rs*2229092, *rs*1041981— were common (MAF ≥ 5%) in at least one population, while the other four “tolerated” polymorphisms were either rare (MAF < 5%) or absent in global populations. The single “deleterious” SNP was present only in the Kinh population from Vietnam at very low frequency (MAF < 0.01). We also determined if common missense variation was conserved across mammalian species using GERP++ [50], phyloP [51] and phastCons [52]. Our results showed that the conservation score for each polymorphic site did not reach the “deleterious” threshold level, in agreement with our SIFT results (Table 2).

**Table 2:** Predictions of amino acid changes on LTα protein function

SNP	Mutation	Damaging Effect	Conservation Score			Protein Stability (ΔΔG)	
		SIFT	phyloP	phastCons	GERP++	I-Mutant3	FoldX
rs2229094	C13R	0.783 (T)	0.065	0.09	1.26	−0.070 (N)	N/A
rs2229092	H51P	0.253 (T)	0.153	0.184	−2.81	0.660 (I)	1.180 (I)
rs1041981	T60N	0.392 (T)	0.073	0.936	3.15	−0.520 (D)	−0.802 (D)

GEPR++, phyloP, and phastCons were applied to the *LTα* coding region to determine if common alleles at *rs*2229094, *rs*2229092 and *rs*1041981 are under evolutionary constraint across mammalian species. The SIFT algorithm was used to predict if a SNP has a (generally negative) effect on protein function. Variants with scores ranging from 0.05 to 1 were considered to be tolerated (T). We also predicted protein stability changes caused by missense mutations at these same three sites—as indicated by ΔΔG— using the FoldX [52] and I-Mutant3 [53] tools (I Increased, D Decreased, N Neutral; ΔΔG is in units of kcal/mol). The corresponding amino acid position, amino acid polymorphisms, and conservation scores are given for each SNP; N/A indicates that ΔΔG could not be calculated for *rs*2229094 using FoldX as outlined in the Methods section

In addition, we analyzed changes in LTα protein stability (i.e. the folded conformation) caused by missense mutations at different common SNPs with two bioinformatic tools: FoldX [53] and I-Mutant3 [54]. Our analyses revealed that the total energy change (ΔΔG) for *rs*1041981, determined by FoldX, was − 0.802 kcal/mol, suggesting that mutation (the A-allele) at this site slightly destabilizes protein structure (Table 2). By contrast, the FoldX ΔΔG for *rs*2229092 was 1.180 kcal/mol, implying that variability at this site largely stabilizes the folding conformation of the LTα protein (Table 2). The I-Mutant3 method yielded similar predictions of protein stability changes due to nucleotide variation at the *rs*1041981 and *rs*2229092 loci. The folding energy difference caused by mutation at *rs*2229094 —calculated using I-Mutant3— was 0.07 kcal/mol (Table 2), indicating that variability at this site has little effect on the folded structure of the LTα protein. Regrettably, we were unable to determine the FoldX ΔΔG for *rs*2229094 because the available template structure, used as input for this bioinformatic tool, did not include residues encoded by this SNP.

Lastly, we examined the potential regulatory effects of variants in or near the *LTα* gene using the SNP2TFBS database [55]. Based on this analysis, we identified 13 non-coding polymorphisms that map to transcription factor binding sites (TFBSs), and are predicted to alter transcription factor binding site affinity (Table 3) [56]. Our data also showed that the transcription factors (TFs) affected by nucleotide changes in the TFBSs were Pdx1, Prrx2, KLF1, ZNF263, SP2, SP1, KLF5, ELF-1, Zfx, Nr1h3/Rxra, Arnt/Ahr, EGR2, Foxd3, FOXI1, IRF1, SPIB, ARID3A. Furthermore, a subset of these 13 SNPs (namely, *rs*2857709, *rs*4947326, *rs*4947327 and *rs*3093542, which are predicted to affect the binding of TFs ELF-1, Zfx and ZNF263) had extreme |*i*HS| and/or |*n*SL| scores, suggesting these candidate functional alleles are or have been targets of recent selection (Table 3; Additional file 2: Table S12). In addition, derived alleles at these sites had coalescence times that ranged from 56,718.38 ya (± 32,106.13 years) to 668,571.54 ya (± 105,795.94 years) (Additional file 2: Table S13). Our analysis also revealed that one of the 13 candidate functional polymorphisms (specifically, *rs*909253)— not inferred to be under selection— is a known genetic risk factor for several complex diseases [10, 13, 57, 58] , and is predicted to alter the binding of TF SPIB (Table 3).

**Table 3:** The effect of allelic variation on transcription factor binding affinity

Genomic coordinate	*rs* identifier	Reference allele	Alternate allele	Genic region	Transcription factor	Selection status	MAF	ClinVar
31,532,814	*rs*2857709	A	G	intergenic	ELF-1	Yes	Common	No
31,533,718	*rs*4947326	A	G	intergenic	Zfx	Yes	Common	No
31,533,722	*rs*113019108	C	T	intergenic	Zfx	No	Common	No
31,533,728	*rs*4947327	G	A	intergenic	Zfx	Yes	Common	No
31,534,206	*rs*2844485	A	G	intergenic	Nr1h3/Rxra	No	Common	No
31,535,455	*rs*549446426	G	A	intergenic	Arnt/Ahr, EGR2	No	Common	No
31,535,459	*rs*62395772	G	A	intergenic	Arnt/Ahr, EGR2	No	Common	No
31,535,462	*rs*538596719	T	C	intergenic	Arnt/Ahr, EGR2	No	Common	No
31,536,796	*rs*2844483	T	G	intergenic	FOXI1, Foxd3	No	Common	No
31,537,221	*rs*62395778	G	A	intergenic	IRF1, ZNF263	No	Common	No
31,540,313	*rs*909253	A	G	intronic	SPIB	No	Common	Yes
31,540,693	*rs*3093542	G	C	intronic	ZNF263	Yes	Common	No
31,541,848	*rs*3093547	T	A	non-coding exon/UTR	ARID3A, Prrx2	No	Common	No

SNP2TFBS [55] was used to map variants to known transcription factor binding sites (TFBSs) in the human genome. In the Table, we have listed the genomic coordinate (from build GRCh 37) of each polymorphic site along with the *rs* identifier, Reference/Alternate alleles, the genic region in which each site is located, as well as the corresponding transcription factor that binds to the TFBSs where polymorphisms occurred. Furthermore, we indicated whether or not a given polymorphic site was inferred to be under selection (Yes/No) and if alleles at this site were common (based on minor allele frequency [MAF]). We also determined if variants were previously reported in the ClinVar database (Yes/No) [57]

## Discussion

### Evidence for soft sweeps in global populations

Our analyses uncovered striking patterns of diversity in *LTα* and the 5′ region in African and non-African populations. In particular, we found significantly positive *D*_T_ and *H* statistics, indicating an excess of intermediate-frequency-derived alleles, within and outside of Africa. Furthermore, we observed the maintenance of multiple high-frequency haplotype lineages in global populations, and inferred a deep coalescent time of variation (> 1.0 million years ago). We also detected low levels of genetic differentiation and extensive sharing of variation among globally diverse populations. A comparative analysis further revealed that a number of polymorphisms were shared between archaic and modern humans (i.e. trans-species polymorphisms). Taken together, these results are largely consistent with a model of long-term balancing selection. However, we also detected long-range EHH on chromosomes carrying derived alleles primarily in non-African populations, and observed a “star-like” phylogeny of haplotypes radiating around common haplotypes in our network. These genetic patterns are consistent with the predictions for positive selection. To reconcile these findings, which appear to support different models of selection, we argue that long-term balancing selection has acted to maintain alleles/haplotypes in African populations, while patterns of diversity and LD in non-Africans were shaped by recent selection.

Within Africa, we found evidence for balancing selection based on the site frequency spectrum and the persistence of multiple common haplotypes in populations. These results are similar to the findings in a prior study that detected signatures of balancing selection at the *TAS2R38* locus—which is associated with normal variation in bitter taste perception—in ethnically diverse African populations [59]. In addition, we detected shorter blocks of EHH in most populations of African descent, likely reflecting a history of recombination in and around *LTα* [60–62]. One exception to this general pattern of LD, however, was observed in African Americans where we found unusually long haplotype structure around the derived allele at *rs*73396237. This SNP is also inferred to be a target of selection and is common in European populations. One possible explanation for this pattern is that the extended LD surrounding this selected SNP could have been introduced into African Americans through gene flow from the ancestors of western Europeans to enslaved West Africans during the Trans-Atlantic slave trade [63]. Indeed, prior studies have shown that contemporary African Americans have varying levels of European ancestry due to this historical event which occurred between the 15th and 19th centuries [63–65]. Additionally, the presence of other derived variation in African Americans (for example, the C-allele at *rs*2229092 in the *LTα* coding region), common in Europeans but notably absent in West Africans, also supports this model of historical admixture. Alternatively, and more provocatively, the common derived allele at *rs*73396237 (inferred to be under selection) could have already been present in the enslaved ancestors of African Americans and then became selectively advantageous in the new environments (for example, novel pathogens) to which these individuals were exposed, leading to extended haplotype structure over time. However, further analysis of a larger set of populations will be needed to distinguish: 1) whether the above extended LD in African Americans was due to strong selection on pre-existing common variation at *rs*73396237 that became beneficial in novel environments, or 2) a selective event had occurred first in the ancestors of Europeans who then admixed with the West African ancestors of present-day African Americans. Interestingly, recent studies have reported instances of populations acquiring selected alleles through past admixture [66–68]. Thus, it is highly conceivable that a selected allele could have been introduced into African Americans through admixture between African and non-African parental populations.

After modern humans migrated from Africa ~ 60,000–80,000 ya, we contend that positive selection on pre-existing genetic variation occurred in regions of the world outside of the African continent. Indeed, this model of adaptation has been reported for several genes in non-African populations [69]. In particular, a recent study uncovered evidence for long-term balancing selection at several loci (*CLCNKB, PKDREJ*, *SDR39U1*, and *ZNF473)* in West and East Africans [69]. However, these genes showed an absence of some of the signals of balancing selection in European and/or East Asian populations; data also indicated that one of the two alleles under balancing selection in Africa underwent a soft sweep in non-Africans [69], likely in response to changes in selective pressures outside of Africa [69–73]. In the present study, we propose that a similar phenomenon occurred in the *LTα* region during the history of non-Africans, leading to the presence of long-range haplotypes in these populations.

Additionally, we suggest that soft sweeps of multiple advantageous mutations occurred— such that pre-existing alleles of similar benefit were selected for and increased in frequency simultaneously, or in short succession of one another (in response to different selective pressures). Under this model of evolution (i.e. a multiple-locus soft sweep model), none of the favored mutations rise rapidly to fixation [74], but rather beneficial alleles at different loci undergo an incomplete selective sweep. In this scenario, multiple mutations can co-exist at intermediate frequency in populations, mimicking balancing selection (resulting in strongly positive *D*_T_ and *H* values) [37, 75–78]. Furthermore, multiple haplotypes carrying selectively advantageous alleles will become frequent within populations, leading to an increase in haplotype diversity [37, 79, 80]. Explicitly, both of these genetic patterns were consistently observed in Eurasian populations. This proposed model of selection is distinct from a classic selective sweep in which a novel beneficial mutation arises once and quickly rises in frequency until it becomes fixed [7].

Lastly, we detected a significant and marginally significant excess of nonsynonymous polymorphisms in African and non-African populations. This bias towards amino acid variation in the *LTα* coding region could arise due to several evolutionary forces, such as positive selection, a relaxation of functional constraint, or balancing selection in diverse populations [60, 81–83]. Although the precise environmental/external pressures driving the different types of selection in and outside of Africa are currently unknown, it is clear that multiple loci in *LTα* and the upstream region are or have been functionally important during human evolution.

### Identification of functional candidate loci

The human genome contains thousands of experimentally corroborated transcription factor binding sites (TFBSs)[84, 85]; precise knowledge of the genetic variants that disrupt TFBSs is critical for understanding the molecular changes that contribute to phenotypic variation [86]. Using a computational approach, we identified a number of SNPs that were predicted to alter the binding of multiple TFs— most notably Zfx, ELF-1, SPIB and ZNF263. Prior studies have also documented the role that these TFs play in gene regulation and immunity. For example, studies have reported that Zfx is essential for the survival of recirculating mature B-cells and embryonic stem cells. Moreover, in vitro experiments demonstrated that Zfx-deficient peripheral T-cells failed to proliferate and expand after microbial antigen stimulation [87–89]. Other studies have also indicated that the ELF-1 binding site is important for the initiation of transcription and that the knockdown of this TF inhibited gene expression [90–92]. Data further showed that TF SPIB plays a role in B-cell differentiation (via gene regulation), enabling these lymphocytes to appropriately respond to environmental cues [93, 94]. In addition, analyses focused on ZNF263 found that this TF can have both positive and negative effects on transcriptional regulation of gene targets; more specifically, in cells where ZNF263 levels were low, a subset (∼15–20%) of genes exhibited decreased transcription, while a larger proportion of genes showed elevated expression [95]. As a result, we suggest that the candidate loci, identified in the present study, likely contribute to phenotypic variation through the regulation of gene expression (though it is not completely clear from our data in which direction these variants affect expression; that is to say, we do not know if they inhibit or enhance expression). Intriguingly, we also found that *rs*909253, which is predicted to alter the binding of SPIB, is associated with increased risk for myocardial infarction, non-Hodgkin lymphoma, and psoriatic arthritis [10, 13, 58], suggesting that the onset of these complex diseases may be due in part to *LTα* expression levels. However, further studies are needed to elucidate the role that *LTα* expression plays in complex disease susceptibility.

Our analyses also revealed strong LD among alleles in and between *LTα* and the 5′ region, raising the possibility that our candidate SNPs are in LD with previously identified variants in the literature. Therefore, it is conceivable that our candidate loci are either causal or act epistatically with previously described alleles in association studies. Furthermore, given the evidence for strong LD between alleles located in different genes (mainly in non-African populations), it is possible that loci in the *LTα* region and in neighboring genes could also interact epistatically, influencing gene expression. Additionally, a subset of the candidate functional sites (i.e., *rs*2857709, *rs*4947326, *rs*4947327, and *rs*3093542) were inferred to be under selection, suggesting that adaptive evolution in the *LTα* region involved changes in the level of gene expression.

Although computational approaches have been beneficial for predicting the impact of nucleotide variation on TF-DNA binding [96–102], the ability to infer functional consequences of nucleotide changes in TFBSs using such methods has its limits. One important limitation to note is that only a small fraction of all existing TFs in the genome have been characterized in terms of their binding properties to date [86]. As a result, a number of TFBSs in the genome do not have known cognate TFs. Equally as important, current computational methods examine each variant in DNA binding sites independently, neglecting the potential epistatic interactions among distinct TFBSs. Therefore, future in vitro and in vivo TF binding experiments will be important for further clarifying how the novel loci in our study singly or jointly, influence function, leading to differences in gene expression.

Our data also indicated that the high levels of amino acid change in the LTα protein is generally tolerated (i.e. they do not disrupt protein function), suggesting that sequence diversity may have a functional purpose. For example, greater amino acid diversity could expand the ability of the *LTα* protein to recognize a broad range of antigens, enhancing immune surveillance. Equally as important, the accumulation of amino acid changes could be critical for the other key functions that the LTα protein performs. While the reasons underlying this bias towards amino acid polymorphisms are not clear, this pattern of diversity has been previously reported for a number of other MHC genes [81]. In addition, our ΔΔG results indicated that the “mutant” proline residue at amino acid position 51— encoded by *rs*2229092— has a stabilizing effect on LTα protein structure relative to the “wild-type” histidine residue at the same site (Table 2). Indeed, it is widely recognized that high stability is important for preserving protein function in a range of conditions over time (for example, temperature and pH) [103]. Furthermore, extra stability is often correlated with protein evolution; specifically, it increases the tolerance of proteins to amino acid substitutions while still folding to its native structure [104]. Thus, highly stable proteins can function across broader physiochemical environments and accept a greater number of amino acid changes, increasing their capacity to perform diverse biochemical tasks [104]. Given that *rs*2229092 was also inferred to be a target of adaptive evolution in non-Africans, we contend that there has been recent selection for nucleotide variability that enhances protein monomer stability, suggesting that the LTα protein may play functionally important and diverse roles in these populations.

Though our analyses of protein structure indicated that nucleotide changes at *rs*2229092, *rs*2229094 and *rs*1041981 were generally tolerated, prior studies have reported that missense mutations at *rs*2229094 and *rs*1041981 are associated with elevated risk for several complex diseases [14, 17, 29]. Given our findings in the present study, we argue that protein instability (leading to dysfunction of the LTα protein) is not likely a major contributor to disease outcome. Further investigation of other possible molecular mechanisms may be more informative for understanding the underlying cause(s) of disease susceptibility (for example, *rs*2229094 and *rs*1041981 may be in LD with other loci that alter the expression of LTα proteins leading to increased risk for disease).

### Timing of common variants in African and non-African populations

Our GENETREE analysis revealed an ancient TMRCA for variation in and near *LTα* (dating to > 1.0 million ya), and a deep coalescence time for common alleles that predate the origin of anatomically modern humans ~ 200,000–300,000 ya [63, 105]. The presence of human polymorphisms in Neanderthal and Denisovan genomes further support the inferred ancient age of common nucleotide variation in our dataset. This sharing of alleles among closely related species suggests that variation in and/or near *LTα* likely arose in modern *H. sapiens* because it was either: 1) inherited from the last ancestor that modern humans shared with Neandertals and Denisovans more than 500,000 ya [106], or 2) introduced into modern humans through admixture with Neanderthal and Denisovan populations who overlapped in space and time with *H. sapiens*. Because we observed these ancient polymorphisms in indigenous African populations, which are known to have low levels of Neandertal/Denisovan ancestry [107, 108], we argue that it is likely these variants originated from the last common ancestor of *H. sapiens*, Neandertals, and Denisovans in Africa rather than through introgression. Regardless, the inferred deep TMRCA of polymorphisms and their presence in closely related hominins suggest that these variants have persisted in the *Homo* lineage for a relatively long period of time.

Lastly, although classical and soft selective sweeps are expected to increase allele frequency differences among populations [109, 110], we did not observe extreme genetic differentiation at common SNPs in and/or near *LTα*. One possible explanation for this pattern is that both ancestral and derived alleles were common in populations in Africa. When modern humans migrated out of Africa within the last 100,000 years [63, 111], they carried ancestral and derived alleles at common sites with them. Because these alleles occur at appreciable frequencies in global populations, it is not surprising that we would not observe large among-population differences in allele frequency and thus not detect unusually large *F*_ST_ values at common sites [112].

### The function and evolution of MHC genes

The MHC is a key component of the adaptive immune system in all jawed vertebrates [113–115]. In humans, MHC is a gene-dense region that spans ∼4  on the short arm of chromosome 6 and contains over 200 genes [113]. The classical MHC class I and class II genes (also called the human leukocyte antigen, HLA, system) encode cell-surface glycoproteins that play a key role in adaptive immunity [114, 115]. In particular, in cells infected by intracellular parasites, MHC class I molecules present parasite-derived peptides to cytotoxic T lymphocytes [114, 115]. Similarly, the MHC class II molecules present antigens (mainly from extracellular pathogens) on the surface of antigen-presenting cells [114, 115]. These exposed peptides are then recognized by helper T lymphocytes, resulting in a series of immune responses [116, 117]. In comparison, proteins encoded by MHC class III genes have somewhat different functions, playing roles in inflammation and complementary immune responses among other immune-related activities [118].

The classical MHC genes are among the most polymorphic in the human genome [114]. Furthermore, alleles at these loci are ancient in age (often predating species divergence events) and can be retained across multiple species. Studies have also indicated that balancing selection has likely acted at these genes to enhance the level of nonsynonymous substitutions in codons and allelic diversity over extremely long time periods [81, 114, 119, 120]. Comparatively, little is known about patterns of variation at genes within the MHC class III region or the microevolutionary forces that have shaped these patterns. However, some studies have identified intergenic sequences in this region that contain *cis*-acting elements for transcriptional or post-transcriptional regulation of gene expression [121, 122]. For example, non-coding sequences upstream of *TNF-α* were found to contain transcription factor and enhancer binding sites that influence *TNF-α* expression in blood serum [122]. In the present study, we similarly identified polymorphisms in the 5′ region of *LTα* that are predicted to alter *LTα* expression levels. However, more in-depth studies of the MHC class III region are needed in order to identify variants with a similar regulatory effect on other structural genes in this chromosomal region, and to better understand the evolutionary history of these genes in the human lineage.

### Concluding remarks

It has been estimated that more than 90% of the SNPs identified in prior association studies lie outside of protein coding genes and that a substantial fraction of these polymorphisms affect gene regulation [123]. Therefore, understanding whether or not variation within TFBSs disrupts or creates functional sites will be informative for elucidating the molecular basis of gene regulation and complex traits, including disease susceptibility. Our study identified SNPs in TFBSs that potentially influence gene expression and were also shared with archaic hominins. The biological consequences of polymorphisms (in modern *H. sapiens*) that originate from closely related species have been a focus of intense scientific inquiry in the field of human genomics [124, 125]. For example, a number of studies have indicated that introgressed Neandertal alleles, occurring at relatively high frequency in contemporary Eurasian populations, contribute to immunity, metabolism, height, hair color, and skin tone [124–136]. Analyses have also shown that introgressed archaic alleles have been targets of positive selection in modern populations. Thus, polymorphisms of non-modern origin are argued to have played important roles in human biology. In the current study, our data suggest that ancient shared alleles in the *LTα* region— likely inherited from the common ancestor of *H. sapiens*, Neandertals, and Denisovans— were functional and adaptive in modern humans. These findings raise new and intriguing questions about whether or not archaic and modern humans exhibited similar phenotypic variation due to their shared allelic variation. Indeed, interrogating the genomes of related species— such as Neandertals and Denisovans— can provide insights into the evolutionary origins of alleles associated with traits that may or may not be unique to modern humans.

## Methods

### Population samples

We analyzed the 9637 bps, encompassing the *LTα* gene (2226 bps) and the adjacent 5′ region (7411 bps) on chromosome 6 in 2039 unrelated individuals from the 1000 Genomes Project (Phase 3). These individuals originated from four geographic regions: [1] Africa includes 99 Esan (ESN, Nigeria), 113 Mandinka (GWD, The Gambia), 54 Mende (MSL, Sierra Leone), 107 Yoruba (YRI, Nigeria), 99 Luhya (LWK, Kenya), 60 African Americans (ASW, United States), 96 Barbadians (ACB, Barbados); [2] South Asia encompasses 84 Bengali (BEB, Bangladesh), 103 Gurjarati (GIH, India), 99 Indian Telugu (ITU, India), 93 Punjabi (PJL, Pakistan), 99 Tamil (STU, Sri Lanka); [3] East Asia consists of 93 Dai (CDX, China), 103 Han (CHB, Beijing), 105 Han (CHS, southern China), 104 Japanese (JPT, Japan), 99 Kinh (KHV, Vietnam); [4] Europe comprise 90 Great British (GBR, Great Britain), 99 Finnish (FIN, Finland), 107 Iberian (IBS, Spain), 104 Toscani Italians (TSI, Italy) [137]. Although we grouped African Americans and African Caribbeans (Barbadians) with indigenous Africans above, we did not combine these recently admixed population with Africans when we examined the geographic distribution of polymorphisms in Fig. 1b and c. As a result, the total number of variants in Fig. 1b and c does not include the population-specific variation present in African Americans and African Caribbeans.

### Nucleotide variation

We extracted variant calls (in vcf files; build GRCh 37 human assembly) from the *LTα* gene and the adjacent 5′ region in the 1000 Genomes sequencing dataset using vcftools [138]. The start and end positions for this region (build GRCh 37) were obtained from NCBI [139]. The minor allele frequency (MAF) was defined as the second most frequent allele at a given site in pooled populations (i.e., 4078 chromosomes). SNPs were also broadly classified as common or rare based on the MAF at a given site. More explicitly, SNPs with an MAF ≥ 5% were classified as common, while SNPs with an MAF < 5% were categorized as rare. For comparative analysis, we examined sequence contigs for ~ 10 kbs, encompassing the *LTα* and 5′ region, in Neandertal and Denisovan samples from the UCSC genome browser [140].

### Tests of neutrality

We calculated the Tajima’s *D* (*D*_T_) [141] and Fay and Wu’s *H* (*H*) [142] statistics for *LTα* and the adjacent 5′ region, separately, in each population. Significance of the test statistics was assessed by comparing the observed values to expected values generated from 10,000 neutral coalescent simulations incorporating different models of growth using the ms software [32]. If a sample showed a statistically significant deviation from the expected theoretical distribution, the null hypothesis of no selection was rejected. For Africans, we calculated expected *D*_T_ and *H* statistics under a 2-, 4-, 6-, 8-, and 10-fold increase in population size (starting from 10,000 individuals) beginning 70,000 ya until the present [34, 143–145]. For non-Africans, we calculated the expected *D*_T_ and *H* values under a range of demographic scenarios that included a population bottleneck at 60,000 ya (from an initial population size of 10,000 individuals and decreasing to 2000 individuals), followed by 10-, 20-, and 40- fold growth beginning at 50,000 ya until the present [144, 145]. For all simulations, we used a generation time of 20 years. Because *LTα* and the 5′ region are different sizes, we also incorporated sequence length as a parameter in these simulations.

In addition, we applied the McDonald–Kreitman (M-K) test to the *LTα* coding region using DnaSP [146]. The M-K test compares the ratio of polymorphism to divergence at replacement and silent sites. Under neutrality, the ratio of replacement to silent substitutions between species (D_N_/D_S_) is expected to equal the ratio of replacement to silent polymorphisms within species (P_N_/P_S_).

### Haplotype variation and inferred relationships

We extracted fully phased haplotype data from the 1000 Genomes Project for 2039 individuals using vcftools [138], and then applied a custom script to identify unique haplotypes along with the number of times that each haplotype appeared in the pooled global dataset. The genealogical relationships among haplotypes were inferred using the median-joining algorithm implemented in the Network 5.0 program [38]. The resulting phylogeny was a tree with the minimum number of changes among all possible trees [38]. Haplotype diversity (*h*-diversity) was also calculated for each population using the DnaSP software [146].

### Linkage disequilibrium

We examined pairwise LD: 1) in the *LTα* region spanning ~ 10 kbs, and separately 2) in an ~ 35.5-kb genomic region, encompassing neighboring genes (*NFKBIL1*, *LTα*, *TNFα*, and *LTβ*), using the Haploview software [40]. LD was quantified using the *D′* statistic [147], which indicates the magnitude of LD between SNP loci based on allele frequency. To maximize our power to detect a relationship between SNP loci, we filtered out SNPs with a MAF < 1% in our analyses. In the resulting LD plots, the color intensity of each square represents the strength of the relationship between SNP alleles. Specifically, bright red squares indicate complete LD between SNP pairs (D′ = 100; logarithm of odds (LOD) > 2); shades of pink/white squares signify little evidence of LD (D′ < 100; LOD < 2); purple squares denote high LD but with little statistical support (low LOD) [40]. The bold triangles in the plots also indicate strong blocks of LD between SNP markers.

### Extended haplotype homozygosity

We characterized long-range LD on chromosome 6 for each population using the *i*HS statistic [42], which indicates the amount of haplotype homozygosity on chromosomes carrying the derived allele compared to chromosomes with the ancestral allele. To identify outlier values, the unstandardized scores for > 340,000 SNPs across the ~ 170-Mb region of chromosome 6 were normalized with the norm program implemented in the selscan package [43]. SNPs with a standardized |*i*HS| > 2 represent the most extreme 5% of scores. We also estimated the length of haplotype homozygosity with another haplotype-based statistic, *n*SL, using selscan [43]. This statistic measures haplotype lengths based on the number of segregating sites in a sample and does not depend on the recombination rate, unlike the *i*HS statistic, making it robust to recombination rate variation [44]. The output results were normalized following the same procedure used for *i*HS. To complement these scans for selection, we quantified the decay of identity of haplotypes with distance by calculating the EHH statistic using loci with extreme *i*HS and/or *n*SL scores as core SNPs [45].

### Population differentiation and structure

To measure the degree of genetic divergence among global populations, we calculated average *F*_ST_ derived from genomic data using the Weir and Cockerham method implemented in vcftools [138, 148]. We also computed among-population *F*_ST_ at individual polymorphic sites across the ~ 10-kb region of interest and at ~ 7.5 million randomly selected SNPs from across the genome. The observed *F*_ST_ estimates for SNPs in and/or near *LTα* were then compared with the empirical distribution of *F*_ST_ values derived from genome-wide SNPs in order to identify outlier values (≤ 2.5th percentile or ≥ 95th percentile of the distribution). *F*_ST_ values at individual sites in or near *LTα,* and *F*_ST_ values at the ~ 7.5 million genome-wide polymorphisms were calculated using the same 21 populations.

### Age estimates of mutations

We used a coalescent-based approach to estimate: 1) the expected TMRCA of the gene tree and 2) the expected ages of individual polymorphisms. This method requires an outgroup that provides the ancestral state at each polymorphic site. We applied the GENETREE software [48] to our sequence data to obtain the maximum likelihood estimate (MLE) of *θ* over 1,000,000 runs [48]. Using the MLE of *θ* and our estimate of *μ*, we calculated the effective population size (*N*_e_) parameter, which was determined to be 18,367 based on the formula *N*_e_ = θ/4μ. Then, the TMRCA of the gene tree and the ages of individual polymorphisms were estimated from the weighted average of simulated ages over 100,000 independent runs [48]. GENETREE assumes an infinite alleles model and no recombination [48]. We removed haplotypes and/or sites that were not compatible with these underlying assumptions before applying the GENETREE algorithm.

### Functional analysis

The effects of nonsynonymous SNPs at *LTα* on protein function were inferred using the SIFT algorithm implemented in the Ensembl Variant Effect Predictor toolset. SIFT predicts the functional impact of amino acid substitutions (i.e., “tolerated” or “deleterious”) based on sequence homology, the physical properties of amino acids and multiple alignment information [49]. In addition to SIFT, we executed GERP++ [49], phyloP [50] and phastCons [51] to evaluate whether or not common missense variation was conserved across 20 different mammalian species [149]. We also predicted changes in LTα protein stability caused by common missense mutations using two bioinformatic tools: FoldX [53] and I-Mutant3 [54] tools. ΔΔ*G* is the difference in the Gibbs free energy for folding between the final state (the mutant) and the reference state (the wild-type). For each polymorphic site, ΔΔG was computed from the free energy of the wild type protein (encoded by the major allele at a given site) minus the Gibbs free energy of the mutated protein (encoded by the minor allele at a given site). In other words, ΔΔG = ΔG WT – ΔG MUT. Given this definition, mutations resulting in negative ΔΔG values were classified as “destabilizing”, while mutations resulting in positive ΔΔG values were categorized as “stabilizing”. We constructed the protein structure of LTα, which served as the input for FoldX, with the homology modelling server SWISS-MODEL [150]. The resulting LTα protein structure only consisted of amino acids from positions 48 to 80, which encompassed *rs*2229092 (H51P) and *rs*1041981 (T60N), but excluded *rs*2229094 (C13R). Therefore, the ΔΔG could not be calculated for *rs*2229094 using the FoldX tool.

We also analyzed polymorphisms in and near the *LTα* gene using the SNP2TFBS tool [55], which predicts if SNPs in TFBSs affect transcription factor binding in the human genome. The effect of a given SNP on transcription factor (TF) binding is estimated based on a position weight matrix (PWM) model for the binding specificity of the corresponding TF factor [55, 56]. In addition, using the NCBI ClinVar database [57], we searched for any reports of an association between variants (inferred to be functional in Table 3) and human health outcomes.

## Supplementary information

**Additional file 1 **: **Figure S1.** contains pairwise LD plots (spanning ~9.64 kbs) for African and non-African populations that were not included in Fig. 3 of the main manuscript. **Figure S2.** contains pairwise LD plots for loci located across a larger genomic region (spanning ~ 35.5 kbs) for each population. **Figure S3.** displays Manhattan plots of standardized |*i*HS| values in populations that were not included in Fig. 4 of the main manuscript. **Figure S4.** shows Manhattan plots of standardized |*nSL*| scores for all populations. **Figure S5.** presents the remaining EHH plots for populations that were not included in Fig. 4 of the main manuscript.

**Additional file 2 **: **Table S1.** contains the frequency of alleles at polymorphic sites across *LTα* and 5′ regulatory region in global populations, and estimates of per site *F*_ST_. **Tables S2.** gives summary statistics for the 5′ regulatory region only. **Table S3.** lists the observed Tajima’s *D* (*D*_T_) statistics for the *LTα* gene and their corresponding *P*-values under different scenarios of population growth. **Table S4.** shows the observed Fay and Wu’s H (*H) * statistics for the *LTα* gene and their corresponding *P*-values under different scenarios of population growth. **Table S5.** lists the observed *D*_T_ statistics for the 5′ regulatory region and their corresponding *P*-values for different scenarios of population growth. **Table S6.** contains the observed *H* statistics for the 5′ regulatory region and their corresponding *P*-values under different scenarios of population growth. **Table S7A.** presents haplotype frequencies in global populations. **Table S7B.** lists the polymorphic sites where the highest-frequency haplotypes in the network differ from one another and also indicates the alleles (on these haplotypes) that are shared with Neandertal and Denisovan genomes. **Table S8.** presents the frequencies of common haplotypes in non-African populations. **Table S9.** shows the frequencies of common haplotypes in African populations. **Table S10.** gives the genomic coordinates for markers in the LD plots (spanning ~9.64 kbs) in Fig. 3 and in Additional file 1: Figure S1. **Table S11.** gives the genomic coordinates for markers in the LD plots (spanning ~35.5 kbs) in Additional file 1: Figure S2. **Table S12.** lists outlier standardized |*i*HS| and |n*SL*| scores for SNPs in each population. **Table S13.** presents the inferred ages of mutations across *LTα* and the 5′ regulatory region.

## Data Availability

The 1000 Genomes Project data (Phase 3) analyzed in the current study are publicly available from an unrestricted online access repository [152].

## Abbreviations

| *i*HS |: Absolute value of the integrated haplotype score
| *n*SL |: Absolute number of segregating sites by length
2*N*: Number of gene copies analyzed in each population
ACB: Barbadians from Barbados
Ahr: Aryl hydrocarbon receptor
ARID3A: AT-Rich Interaction Domain 3A
Arnt: Aryl hydrocarbon receptor nuclear translocator)
ASW: African Americans from the Southwest United States
BEB: Bengali from Bangladesh
bp: base pair
bps: base pairs
C13R: Cysteine/Arginine polymorphism at amino acid position 13
CDX: Chinese Dai from Xishuangbanna, China
CHB: Han Chinese from Beijing
CHS: Han Chinese from southern China
*CLCNKB*: Chloride Voltage-gated Channel Kb (gene)
D_N_: Replacement divergent sites
DNA: Deoxyribonucleic Acid
D_S_: Silent divergent sites
D_*T*_: Tajima’s *D*
EGR2: Early Growth Response 2
EHH: Extended haplotype homozygosity
ELF-1: E74 like ETS transcription factor 1
ESN: Esan from Nigeria
FIN: Finnish from Finland
Foxd3: Forkhead Box D3
FOXI1: Forkhead Box I1
*F*_ST_: Fixation index (subpopulation relative to total population)
GBR: Great British from the United Kingdom
GIH: Gujarati Indian from Houston, Texas
GRCh37: Genome Reference Consortium Human Build 37
GWD: Gambian in Western Divisions in the Gambia
*H*: Fay and Wu’s *H*
h: Number of haplotypes
*H. sapiens*: *Homo sapiens*
H51P: Histidine/Proline polymorphism at amino acid position 51
*h*-diversity: Haplotype diversity
HLA: Human Leukocyte Antigen
IBS: Iberian population in Spain
*i*HS: integrated haplotype score
IRF1: Interferon Regulatory Factor 1
ITU: Indian Telugu from the UK
JPT: Japanese in Tokyo, Japan
kb: kilobase
kbs: kilobases
kcal/mol: one kilocalorie of energy per one mole of substance
KHV: Kinh in Ho Chi Minh City, Vietnam
KLF1: Kruppel Like Factor 1
KLF5: Kruppel Like Factor 5
LD: Linkage disequilibrium
LOD: Logarithm of odds
*LTα*: Lymphotoxin alpha (gene)
*LTβ*: Lymphotoxin beta (gene)
LWK: Luhya in Webuye, Kenya
MAF: Minor Allele Frequency
Mb: Megabase
Mbs: Megabases
MHC: Major Histocompatibility Complex
M-K: McDonald-Kreitman
MLE: Maximum Likelihood Estimate
MSL: Mende in Sierra Leone
NCBI: National Center for Biotechnology Information
*N*_e_: Effective population size
*NFKBIL1*: NFKB inhibitor like 1 (gene)
NK/T-cell: Natural killer T-cell
Nr1h3: Nuclear Receptor Subfamily 1 Group H Member 3
*n*SL: number of segregating sites by length
Pdx1: Pancreas/Duodenum Homeobox Protein 1
pH: potential Hydrogen
PJL: Punjabi from Lahore, Pakistan
*PKDREJ*: Polycystin family receptor for egg jelly (gene)
P_N_: Replacement polymorphic sites
Prrx2: Paired Related Homeobox 2
P_S_: Silent polymorphic sites
PWM: Position Weight Matrix
RxrA: Retinoid X Receptor Alpha
*S*: Number of segregating sites
*SDR39U1*: Short chain dehydrogenase/reductase family 39 U member 1 (gene)
SNP: Single nucleotide polymorphism
SNPs: Single nucleotide polymorphisms
SP1: Specificity proteins 1
SP2: Specificity proteins 2
SPIB: Spi-B transcription factor
STU: Sri Lankan Tamil from the UK
T60N: Threonine/Asparagine polymorphism at amino acid position 60
*TAS2R38*: Taste 2 receptor member 38 (gene)
TF: Transcription Factor
TFBSs: Transcription Factor Binding Sites
TF-DNA: Transcription Factor-Deoxyribonucleic Acid
TFs: Transcription Factors
TMRCA: Time to the most recent common ancestor
*TNFα*: Tumor necrosis factor alpha (gene)
TSI: Toscani in Italy
UCSC: University of California Santa Cruz
UTR: Untranslated region
vcf: variant call format
ya: years ago
YRI: Yoruba in Ibadan, Nigeria
Zfx: Zinc finger X-chromosomal protein
ZNF263: Zinc Finger Protein 263
ZNF473: Zinc Finger Protein 473
ΔG MUT: DeltaG for the mutant allele
ΔG WT: DeltaG for the wild-type allele
ΔΔG: DeltaDeltaG (the total energy change)
*θ*: *Scaled* population *mutation rate*
*θ*_W_: Watterson’s theta (nucleotide diversity per site)
*θ*_π_: Mean pairwise number of differences between sequences per site
*μ*: Mutation rate per sequence per generation

## References

[1] Bauer J, Namineni S, Reisinger F, Zoller J, Yuan D, Heikenwalder M (2012). Lymphotoxin, NF-kB, and cancer: the dark side of cytokines. Dig Dis.

[2] Daller B, Musch W, Rohrl J, Tumanov AV, Nedospasov SA, Mannel DN (2011). Lymphotoxin-beta receptor activation by lymphotoxin-alpha(1)beta(2) and LIGHT promotes tumor growth in an NFkappaB-dependent manner. Int J Cancer.

[3] Etemadi N, Holien JK, Chau D, Dewson G, Murphy JM, Alexander WS (2013). Lymphotoxin alpha induces apoptosis, necroptosis and inflammatory signals with the same potency as tumour necrosis factor. FEBS J.

[4] Etemadi N, Webb A, Bankovacki A, Silke J, Nachbur U (2013). Progranulin does not inhibit TNF and lymphotoxin-alpha signalling through TNF receptor 1. Immunol Cell Biol.

[5] Liu J, Liu J, Song B, Wang T, Liu Y, Hao J (2013). Genetic variations in CTLA-4, TNF-alpha, and LTA and susceptibility to T-cell lymphoma in a Chinese population. Cancer Epidemiol.

[6] Yu X, Huang Y, Li C, Yang H, Lu C, Duan S (2014). Positive association between lymphotoxin-alpha variation rs909253 and cancer risk: a meta-analysis based on 36 case-control studies. Tumour Biol.

[7] Messer G, Spengler U, Jung MC, Honold G, Blomer K, Pape GR (1991). Polymorphic structure of the tumor necrosis factor (TNF) locus: an NcoI polymorphism in the first intron of the human TNF-beta gene correlates with a variant amino acid in position 26 and a reduced level of TNF-beta production. J Exp Med.

[8] Tan JH, Temple SE, Kee C, Waterer GW, Tan CR, Gut I (2011). Characterisation of TNF block haplotypes affecting the production of TNF and LTA. Tissue Antigens.

[9] Yokley BH, Selby ST, Posch PE (2013). A stimulation-dependent alternate core promoter links lymphotoxin alpha expression with TGF-beta1 and fibroblast growth factor-7 signaling in primary human T cells. J Immunol.

[10] Ozaki K, Ohnishi Y, Iida A, Sekine A, Yamada R, Tsunoda T (2002). Functional SNPs in the lymphotoxin-alpha gene that are associated with susceptibility to myocardial infarction. Nat Genet.

[11] Knight JC, Keating BJ, Kwiatkowski DP (2004). Allele-specific repression of lymphotoxin-alpha by activated B cell factor-1. Nat Genet.

[12] Taylor JM, Wicks K, Vandiedonck C, Knight JC (2008). Chromatin profiling across the human tumour necrosis factor gene locus reveals a complex, cell type-specific landscape with novel regulatory elements. Nucleic Acids Res.

[13] Cheng S, Li J, Liu W, Liu C, Su L, Liu X (2015). LTA + 252A > G polymorphism is associated with risk of nasal NK/T-cell lymphoma in a Chinese population: a case-control study. BMC Cancer.

[14] Huang Y, Yu X, Wang L, Zhou S, Sun J, Feng N (2013). Four genetic polymorphisms of lymphotoxin-alpha gene and cancer risk: a systematic review and meta-analysis. PLoS One.

[15] Lu R, Dou X, Gao X, Zhang J, Ni J, Guo L (2012). A functional polymorphism of lymphotoxin-alpha (LTA) gene rs909253 is associated with gastric cancer risk in an Asian population. Cancer Epidemiol.

[16] Zhou P, Huang W, Chu X, Du LF, Li JP, Zhang C (2012). The lymphotoxin-alpha 252A>G polymorphism and breast cancer: a meta-analysis. Asian Pac J Cancer Prev.

[17] Sainz J, Rudolph A, Hoffmeister M, Frank B, Brenner H, Chang-Claude J (2012). Effect of type 2 diabetes predisposing genetic variants on colorectal cancer risk. J Clin Endocrinol Metab.

[18] Skibola CF, Bracci PM, Nieters A, Brooks-Wilson A, de Sanjose S, Hughes AM (2010). Tumor necrosis factor (TNF) and lymphotoxin-alpha (LTA) polymorphisms and risk of non-Hodgkin lymphoma in the InterLymph Consortium. Am J Epidemiol.

[19] Wang SS, Purdue MP, Cerhan JR, Zheng T, Menashe I, Armstrong BK (2009). Common gene variants in the tumor necrosis factor (TNF) and TNF receptor superfamilies and NF-kB transcription factors and non-Hodgkin lymphoma risk. PLoS One.

[20] Aissani B, Ogwaro KM, Shrestha S, Tang J, Breen EC, Wong HL (2009). The major histocompatibility complex conserved extended haplotype 8.1 in AIDS-related non-Hodgkin lymphoma. J Acquir Immune Defic Syndr.

[21] Haybaeck J, Zeller N, Wolf MJ, Weber A, Wagner U, Kurrer MO (2009). A lymphotoxin-driven pathway to hepatocellular carcinoma. Cancer Cell.

[22] Ramasawmy R, Fae KC, Cunha-Neto E, Muller NG, Cavalcanti VL, Ferreira RC (2007). Polymorphisms in the gene for lymphotoxin-alpha predispose to chronic Chagas cardiomyopathy. J Infect Dis.

[23] Iwanaga Y, Ono K, Takagi S, Terashima M, Tsutsumi Y, Mannami T (2004). Association analysis between polymorphisms of the lymphotoxin-alpha gene and myocardial infarction in a Japanese population. Atherosclerosis.

[24] Consortium P (2004). A trio family study showing association of the lymphotoxin-alpha N26 (804A) allele with coronary artery disease. Eur J Hum Genet.

[25] Fassmann A, Holla LI, Buckova D, Vasku A, Znojil V, Vanek J (2003). Polymorphisms in the +252(a/G) lymphotoxin-alpha and the −308(a/G) tumor necrosis factor-alpha genes and susceptibility to chronic periodontitis in a Czech population. J Periodontal Res.

[26] Jia B, Qi X (2017). The genetic association between polymorphisms in lymphotoxin-alpha gene and ankylosing spondylitis susceptibility in Chinese group: a case-control study. Medicine (Baltimore).

[27] Saad MN, Mabrouk MS, Eldeib AM, Shaker OG (2015). Genetic case-control study for eight polymorphisms associated with rheumatoid arthritis. PLoS One.

[28] Zhang C, Zhao MQ, Liu J, Huang Q, Li P, Ni J (2015). Association of lymphotoxin alpha polymorphism with systemic lupus erythematosus and rheumatoid arthritis: a meta-analysis. Int J Rheum Dis.

[29] Laddha NC, Dwivedi M, Gani AR, Mansuri MS, Begum R (2013). Tumor necrosis factor B (TNFB) genetic variants and its increased expression are associated with vitiligo susceptibility. PLoS One.

[30] Bolstad AI, Le Hellard S, Kristjansdottir G, Vasaitis L, Kvarnstrom M, Sjowall C (2012). Association between genetic variants in the tumour necrosis factor/lymphotoxin alpha/lymphotoxin beta locus and primary Sjogren's syndrome in Scandinavian samples. Ann Rheum Dis.

[31] Phillips CM, Goumidi L, Bertrais S, Ferguson JF, Field MR, Kelly ED (2010). Additive effect of polymorphisms in the IL-6, LTA, and TNF-{alpha} genes and plasma fatty acid level modulate risk for the metabolic syndrome and its components. J Clin Endocrinol Metab.

[32] Hudson RR (2002). Generating samples under a Wright-fisher neutral model of genetic variation. Bioinformatics.

[33] Wall JD, Przeworski M (2000). When did the human population size start increasing?. Genetics.

[34] Cox MP, Morales DA, Woerner AE, Sozanski J, Wall JD, Hammer MF (2009). Autosomal resequence data reveal late stone age signals of population expansion in sub-Saharan African foraging and farming populations. PLoS One.

[35] Ferrer-Admetlla A, Bosch E, Sikora M, Marques-Bonet T, Ramirez-Soriano A, Muntasell A (2008). Balancing selection is the main force shaping the evolution of innate immunity genes. J Immunol.

[36] Hancock AM, Rienzo AD (2008). Detecting the genetic signature of natural selection in human populations: models, methods, and data. Annu Rev Anthropol.

[37] Przeworski M, Coop G, Wall JD (2005). The signature of positive selection on standing genetic variation. Evolution.

[38] Bandelt HJ, Forster P, Rohl A (1999). Median-joining networks for inferring intraspecific phylogenies. Mol Biol Evol.

[39] Bamshad M, Wooding SP (2003). Signatures of natural selection in the human genome. Nat Rev Genet.

[40] Barrett JC, Fry B, Maller J, Daly MJ (2005). Haploview: analysis and visualization of LD and haplotype maps. Bioinformatics.

[41] Posada D, Crandall KA (2001). Intraspecific gene genealogies: trees grafting into networks. Trends Ecol Evol.

[42] Voight BF, Kudaravalli S, Wen X, Pritchard JK (2006). A map of recent positive selection in the human genome. PLoS Biol.

[43] Szpiech ZA, Hernandez RD (2014). Selscan: an efficient multithreaded program to perform EHH-based scans for positive selection. Mol Biol Evol.

[44] Ferrer-Admetlla A, Liang M, Korneliussen T, Nielsen R (2014). On detecting incomplete soft or hard selective sweeps using haplotype structure. Mol Biol Evol.

[45] Sabeti PC, Reich DE, Higgins JM, Levine HZ, Richter DJ, Schaffner SF (2002). Detecting recent positive selection in the human genome from haplotype structure. Nature.

[46] Campbell MC, Tishkoff SA (2008). African genetic diversity: implications for human demographic history, modern human origins, and complex disease mapping. Annu Rev Genomics Hum Genet.

[47] Elhaik E (2012). Empirical distributions of F (ST) from large-scale human polymorphism data. PLoS One.

[48] Griffiths RC, Tavaré S (1994). Ancestral inference in population genetics. Stat Sci.

[49] Sim NL, Kumar P, Hu J, Henikoff S, Schneider G, Ng PC (2012). SIFT web server: predicting effects of amino acid substitutions on proteins. Nucleic Acids Res.

[50] Davydov EV, Goode DL, Sirota M, Cooper GM, Sidow A, Batzoglou S (2010). Identifying a high fraction of the human genome to be under selective constraint using GERP++. PLoS Comput Biol.

[51] Pollard KS, Hubisz MJ, Rosenbloom KR, Siepel A (2010). Detection of nonneutral substitution rates on mammalian phylogenies. Genome Res.

[52] Siepel A, Bejerano G, Pedersen JS, Hinrichs AS, Hou M, Rosenbloom K (2005). Evolutionarily conserved elements in vertebrate, insect, worm, and yeast genomes. Genome Res.

[53] Schymkowitz J, Borg J, Stricher F, Nys R, Rousseau F, Serrano L (2005). The FoldX web server: an online force field. Nucleic Acids Res.

[54] Capriotti E, Fariselli P, Casadio R (2005). I-Mutant2.0: predicting stability changes upon mutation from the protein sequence or structure. Nucleic Acids Res.

[55] Kumar S, Ambrosini G, Bucher P (2017). SNP2TFBS - a database of regulatory SNPs affecting predicted transcription factor binding site affinity. Nucleic Acids Res.

[56] Mathelier Anthony, Fornes Oriol, Arenillas David J., Chen Chih-yu, Denay Grégoire, Lee Jessica, Shi Wenqiang, Shyr Casper, Tan Ge, Worsley-Hunt Rebecca, Zhang Allen W., Parcy François, Lenhard Boris, Sandelin Albin, Wasserman Wyeth W. (2015). JASPAR 2016: a major expansion and update of the open-access database of transcription factor binding profiles. Nucleic Acids Research.

[57] Landrum Melissa J, Lee Jennifer M, Benson Mark, Brown Garth R, Chao Chen, Chitipiralla Shanmuga, Gu Baoshan, Hart Jennifer, Hoffman Douglas, Jang Wonhee, Karapetyan Karen, Katz Kenneth, Liu Chunlei, Maddipatla Zenith, Malheiro Adriana, McDaniel Kurt, Ovetsky Michael, Riley George, Zhou George, Holmes J Bradley, Kattman Brandi L, Maglott Donna R (2017). ClinVar: improving access to variant interpretations and supporting evidence. Nucleic Acids Research.

[58] Balding Joanna, Kane David, Livingstone Wendy, Mynett-Johnson Lesley, Bresnihan Barry, Smith Owen, FitzGerald Oliver (2003). Cytokine gene polymorphisms: Association with psoriatic arthritis susceptibility and severity. Arthritis & Rheumatism.

[59] Campbell M. C., Ranciaro A., Froment A., Hirbo J., Omar S., Bodo J.-M., Nyambo T., Lema G., Zinshteyn D., Drayna D., Breslin P. A. S., Tishkoff S. A. (2011). Evolution of Functionally Diverse Alleles Associated with PTC Bitter Taste Sensitivity in Africa. Molecular Biology and Evolution.

[60] Charlesworth Deborah (2006). Balancing Selection and Its Effects on Sequences in Nearby Genome Regions. PLoS Genetics.

[61] Charlesworth B, Nordborg M, Charlesworth D (1997). The effects of local selection, balanced polymorphism and background selection on equilibrium patterns of genetic diversity in subdivided populations. Genet Res.

[62] Messer PW, Petrov DA (2013). Population genomics of rapid adaptation by soft selective sweeps. Trends Ecol Evol.

[63] Campbell MC, Hirbo JB, Townsend JP, Tishkoff SA (2014). The peopling of the African continent and the diaspora into the new world. Curr Opin Genet Dev.

[64] Bryc K, Auton A, Nelson MR, Oksenberg JR, Hauser SL, Williams S (2010). Genome-wide patterns of population structure and admixture in west Africans and African Americans. Proc Natl Acad Sci U S A.

[65] Bryc K, Durand EY, Macpherson JM, Reich D, Mountain JL (2015). The genetic ancestry of African Americans, Latinos, and European Americans across the United States. Am J Hum Genet.

[66] Laso-Jadart R, Harmant C, Quach H, Zidane N, Tyler-Smith C, Mehdi Q (2017). The genetic legacy of the Indian Ocean slave trade: recent admixture and post-admixture selection in the Makranis of Pakistan. Am J Hum Genet.

[67] Pierron D, Heiske M, Razafindrazaka H, Pereda-Loth V, Sanchez J, Alva O (2018). Strong selection during the last millennium for African ancestry in the admixed population of Madagascar. Nat Commun.

[68] Ranciaro A, Campbell MC, Hirbo JB, Ko WY, Froment A, Anagnostou P (2014). Genetic origins of lactase persistence and the spread of pastoralism in Africa. Am J Hum Genet.

[69] de Filippo C, Key FM, Ghirotto S, Benazzo A, Meneu JR, Weihmann A (2016). Recent selection changes in human genes under long-term balancing selection. Mol Biol Evol.

[70] Coop G, Pickrell JK, Novembre J, Kudaravalli S, Li J, Absher D (2009). The role of geography in human adaptation. PLoS Genet.

[71] Fumagalli M, Sironi M, Pozzoli U, Ferrer-Admetlla A, Pattini L, Nielsen R (2011). Signatures of environmental genetic adaptation pinpoint pathogens as the main selective pressure through human evolution. PLoS Genet.

[72] Gravel S, Henn BM, Gutenkunst RN, Indap AR, Marth GT, Clark AG (2011). Demographic history and rare allele sharing among human populations. Proc Natl Acad Sci U S A.

[73] Pickrell JK, Coop G, Novembre J, Kudaravalli S, Li JZ, Absher D (2009). Signals of recent positive selection in a worldwide sample of human populations. Genome Res.

[74] Pritchard JK, Pickrell JK, Coop G (2010). The genetics of human adaptation: hard sweeps, soft sweeps, and polygenic adaptation. Curr Biol.

[75] Jensen JD (2014). On the unfounded enthusiasm for soft selective sweeps. Nat Commun.

[76] Hermisson J, Pennings PS (2017). Soft sweeps and beyond: understanding the patterns and probabilities of selection footprints under rapid adaptation. Methods Ecol Evol.

[77] Barrett RD, Schluter D (2008). Adaptation from standing genetic variation. Trends Ecol Evol.

[78] Croze M, Zivkovic D, Stephan W, Hutter S (2016). Balancing selection on immunity genes: review of the current literature and new analysis in Drosophila melanogaster. Zoology (Jena).

[79] Teshima KM, Coop G, Przeworski M (2006). How reliable are empirical genomic scans for selective sweeps?. Genome Res.

[80] Smith JM, Haigh J (1974). The hitch-hiking effect of a favourable gene. Genet Res.

[81] Pierini Federica, Lenz Tobias L (2018). Divergent Allele Advantage at Human MHC Genes: Signatures of Past and Ongoing Selection. Molecular Biology and Evolution.

[82] Gillespie JH (1991). The causes of molecular evolution.

[83] Engle EK, Fay JC (2013). ZRT1 Harbors an Excess of Nonsynonymous Polymorphism and Shows Evidence of Balancing Selection in *Saccharomyces cerevisiae*. G3 (Bethesda).

[84] Levitsky VG, Kulakovskiy IV, Ershov NI, Oshchepkov DY, Makeev VJ, Hodgman TC (2014). Application of experimentally verified transcription factor binding sites models for computational analysis of ChIP-Seq data. BMC Genomics.

[85] Vorontsov IE, Fedorova AD, Yevshin IS, Sharipov RN, Kolpakov FA, Makeev VJ (2018). Genome-wide map of human and mouse transcription factor binding sites aggregated from ChIP-Seq data. BMC Res Notes.

[86] Deplancke B, Alpern D, Gardeux V (2016). The genetics of transcription factor DNA binding variation. Cell.

[87] Arenzana TL, Smith-Raska MR, Reizis B (2009). Transcription factor Zfx controls BCR-induced proliferation and survival of B lymphocytes. Blood.

[88] Galan-Caridad JM, Harel S, Arenzana TL, Hou ZE, Doetsch FK, Mirny LA (2007). Zfx controls the self-renewal of embryonic and hematopoietic stem cells. Cell.

[89] Smith-Raska MR, Arenzana TL, D’Cruz LM, Khodadadi-Jamayran A, Tsirigos A, Goldrath AW (2018). The transcription factor Zfx regulates peripheral T cell self-renewal and proliferation. Front Immunol.

[90] Nishiyama C, Yokota T, Okumura K, Ra C (1999). The transcription factors elf-1 and GATA-1 bind to cell-specific enhancer elements of human high-affinity IgE receptor alpha-chain gene. J Immunol.

[91] Tsokos GC, Nambiar MP, Juang YT (2003). Activation of the Ets transcription factor elf-1 requires phosphorylation and glycosylation: defective expression of activated elf-1 is involved in the decreased TCR zeta chain gene expression in patients with systemic lupus erythematosus. Ann N Y Acad Sci.

[92] Yang J, Yang W, Hirankarn N, Ye DQ, Zhang Y, Pan HF (2011). ELF1 is associated with systemic lupus erythematosus in Asian populations. Hum Mol Genet.

[93] Willis SN, Tellier J, Liao Y, Trezise S, Light A, O’Donnell K (2017). Environmental sensing by mature B cells is controlled by the transcription factors PU.1 and SpiB. Nat Commun.

[94] DeKoter RP, Geadah M, Khoosal S, Xu LS, Thillainadesan G, Torchia J (2010). Regulation of follicular B cell differentiation by the related E26 transformation-specific transcription factors PU.1, Spi-B, and Spi-C. J Immunol.

[95] Frietze S, Lan X, Jin VX, Farnham PJ (2010). Genomic targets of the KRAB and SCAN domain-containing zinc finger protein 263. J Biol Chem.

[96] Barrera LA, Vedenko A, Kurland JV, Rogers JM, Gisselbrecht SS, Rossin EJ (2016). Survey of variation in human transcription factors reveals prevalent DNA binding changes. Science.

[97] Boyle AP, Hong EL, Hariharan M, Cheng Y, Schaub MA, Kasowski M (2012). Annotation of functional variation in personal genomes using RegulomeDB. Genome Res.

[98] Coetzee SG, Shen HC, Hazelett DJ, Lawrenson K, Kuchenbaecker K, Tyrer J (2015). Cell-type-specific enrichment of risk-associated regulatory elements at ovarian cancer susceptibility loci. Hum Mol Genet.

[99] Lambert SA, Jolma A, Campitelli LF, Das PK, Yin Y, Albu M (2018). The human transcription factors. Cell.

[100] Lee D, Gorkin DU, Baker M, Strober BJ, Asoni AL, McCallion AS (2015). A method to predict the impact of regulatory variants from DNA sequence. Nat Genet.

[101] Zhao J, Li D, Seo J, Allen AS, Gordan R (2017). Quantifying the impact of non-coding variants on transcription factor-DNA binding. Res Comput Mol Biol.

[102] Mathelier A, Wasserman WW (2013). The next generation of transcription factor binding site prediction. PLoS Comput Biol.

[103] Deller MC, Kong L, Rupp B (2016). Protein stability: a crystallographer’s perspective. Acta Crystallogr F Struct Biol Commun.

[104] Bloom JD, Labthavikul ST, Otey CR, Arnold FH (2006). Protein stability promotes evolvability. Proc Natl Acad Sci U S A.

[105] Hublin JJ, Ben-Ncer A, Bailey SE, Freidline SE, Neubauer S, Skinner MM (2017). New fossils from Jebel Irhoud, Morocco and the pan-African origin of Homo sapiens. Nature.

[106] Green RE, Krause J, Briggs AW, Maricic T, Stenzel U, Kircher M (2010). A draft sequence of the Neandertal genome. Science.

[107] Povysil G, Hochreiter S (2016). IBD sharing between Africans, Neandertals, and Denisovans. Genome Biol Evol.

[108] Higham T, Douka K, Wood R, Ramsey CB, Brock F, Basell L (2014). The timing and spatiotemporal patterning of Neanderthal disappearance. Nature.

[109] Hohenlohe PA, Phillips PC, Cresko WA (2010). Using population genomics to detect selection in natural populations: Key concepts and methodological considerations. Int J Plant Sci.

[110] Peter BM, Huerta-Sanchez E, Nielsen R (2012). Distinguishing between selective sweeps from standing variation and from a de novo mutation. PLoS Genet.

[111] Pagani L, Schiffels S, Gurdasani D, Danecek P, Scally A, Chen Y (2015). Tracing the route of modern humans out of Africa by using 225 human genome sequences from Ethiopians and Egyptians. Am J Hum Genet.

[112] Campbell MC, Smith LT, Harvey J (2019). Population genetic evidence for positive and purifying selection acting at the human IFN-γ locus in Africa. Genes Immun.

[113] Beck S, Trowsdale J (2000). The human major histocompatability complex: lessons from the DNA sequence. Annu Rev Genomics Hum Genet.

[114] Trowsdale J (2011). The MHC, disease and selection. Immunol Lett.

[115] Trowsdale J, Knight JC (2013). Major histocompatibility complex genomics and human disease. Annu Rev Genomics Hum Genet.

[116] Jensen PE (2007). Recent advances in antigen processing and presentation. Nat Immunol.

[117] Neefjes J, Jongsma ML, Paul P, Bakke O (2011). Towards a systems understanding of MHC class I and MHC class II antigen presentation. Nat Rev Immunol.

[118] Deakin JE, Papenfuss AT, Belov K, Cross JG, Coggill P, Palmer S (2006). Evolution and comparative analysis of the MHC class III inflammatory region. BMC Genomics.

[119] Lenz TL (2018). Adaptive value of novel MHC immune gene variants. Proc Natl Acad Sci U S A.

[120] Milner CM, Campbell RD (2001). Genetic organization of the human MHC class III region. Front Biosci.

[121] Yung Yu C, Yang Z, Blanchong CA, Miller W (2000). The human and mouse MHC class III region: a parade of 21 genes at the centromeric segment. Immunol Today.

[122] Qidwai T, Khan F (2011). Tumour necrosis factor gene polymorphism and disease prevalence. Scand J Immunol.

[123] Hrdlickova B, de Almeida RC, Borek Z, Withoff S (2014). Genetic variation in the non-coding genome: involvement of micro-RNAs and long non-coding RNAs in disease. Biochim Biophys Acta.

[124] Racimo F, Sankararaman S, Nielsen R, Huerta-Sanchez E (2015). Evidence for archaic adaptive introgression in humans. Nat Rev Genet.

[125] Vattathil S, Akey JM (2015). Small amounts of archaic admixture provide big insights into human history. Cell.

[126] Dannemann M, Kelso J (2017). The contribution of Neanderthals to phenotypic variation in modern humans. Am J Hum Genet.

[127] Dolgova O, Lao O (2018). Evolutionary and Medical Consequences of Archaic Introgression into Modern Human Genomes. Genes (Basel).

[128] Racimo F, Gokhman D, Fumagalli M, Ko A, Hansen T, Moltke I (2017). Archaic adaptive introgression in TBX15/WARS2. Mol Biol Evol.

[129] Abi-Rached L, Jobin MJ, Kulkarni S, McWhinnie A, Dalva K, Gragert L (2011). The shaping of modern human immune systems by multiregional admixture with archaic humans. Science.

[130] Dannemann M, Andres AM, Kelso J (2016). Introgression of Neandertal- and Denisovan-like haplotypes contributes to adaptive variation in human toll-like receptors. Am J Hum Genet.

[131] Huerta-Sanchez E, Jin X, Asan, Bianba Z, Peter BM, Vinckenbosch N (2014). Altitude adaptation in Tibetans caused by introgression of Denisovan-like DNA. Nature.

[132] Sankararaman S, Mallick S, Dannemann M, Prufer K, Kelso J, Paabo S (2014). The genomic landscape of Neanderthal ancestry in present-day humans. Nature.

[133] Vernot B, Akey JM (2014). Resurrecting surviving Neandertal lineages from modern human genomes. Science.

[134] Deschamps M, Laval G, Fagny M, Itan Y, Abel L, Casanova JL (2016). Genomic signatures of selective pressures and introgression from archaic hominins at human innate immunity genes. Am J Hum Genet.

[135] Dannemann M, Racimo F (2018). Something old, something borrowed: admixture and adaptation in human evolution. Curr Opin Genet Dev.

[136] Marciniak S, Perry GH (2017). Harnessing ancient genomes to study the history of human adaptation. Nat Rev Genet.

[137] The 1000 Genomes Project Consortium, Abecasis GR, Auton A, Brooks LD, DePristo MA, Durbin RM, et al. An integrated map of genetic variation from 1,092 human genomes. Nature. 2012;491(7422):56–65. 10.1038/nature11632 PMC3498066 23128226

[138] Danecek P, Auton A, Abecasis G, Albers CA, Banks E, DePristo MA (2011). The variant call format and VCFtools. Bioinformatics.

[139] National Library of Medicine (US). Cited January 26, 2019. Available from: https://www.ncbi.nlm.nih.gov/ (Accessed 27 Jan 2019).

[140] Kent WJ, Sugnet CW, Furey TS, Roskin KM, Pringle TH, Zahler AM (2002). The human genome browser at UCSC. Genome Res.

[141] Tajima F, Nei M (1984). Estimation of evolutionary distance between nucleotide sequences. Mol Biol Evol.

[142] Fay JC, Wu CI (2000). Hitchhiking under positive Darwinian selection. Genetics.

[143] Gay J, Myers S, McVean G (2007). Estimating meiotic gene conversion rates from population genetic data. Genetics.

[144] Marth GT, Czabarka E, Murvai J, Sherry ST (2004). The allele frequency spectrum in genome-wide human variation data reveals signals of differential demographic history in three large world populations. Genetics.

[145] Voight BF, Adams AM, Frisse LA, Qian Y, Hudson RR, Di Rienzo A (2005). Interrogating multiple aspects of variation in a full resequencing data set to infer human population size changes. Proc Natl Acad Sci U S A.

[146] Librado P, Rozas J (2009). DnaSP v5: a software for comprehensive analysis of DNA polymorphism data. Bioinformatics.

[147] Lewontin RC (1964). The interaction of selection and linkage. I. General Considerations; Heterotic Models. Genetics.

[148] Weir BS, Cockerham CC (1984). Estimating F-statistics for the analysis of population structure. Evolution.

[149] The UCSC Genome Browser database. Cited January 27, 2019. Available from: http://www.noncode.org/cgi-bin/hgTables?db=hg38&hgta_group=compGeno&hgta_track=cons20way&hgta_table=multiz20way&hgta_doSchema=describe+table+schema. Accessed 27 Jan 2019.

[150] Biasini M, Bienert S, Waterhouse A, Arnold K, Studer G, Schmidt T (2014). SWISS-MODEL: modelling protein tertiary and quaternary structure using evolutionary information. Nucleic Acids Res.

[151] IGSR: The International Genome Sample Resource. Available from: https://www.internationalgenome.org/sample_collection_principles/. (Accessed 27 Jan 2019)

[152] 1000 Genomes Project. Cited January 27, 2019. Available from: ftp://ftptrace.ncbi.nih.gov/1000genomes/ftp/release/20130502 (Accessed 27 Jan 2019).

