# Correction to: Multiple selective sweeps of ancient polymorphisms in and around LTα located in the MHC class III region on chromosome 6

**DOI:** 10.1186/s12862-019-1557-2

**Published:** 2019-12-16

**Authors:** Michael C. Campbell, Bryan Ashong, Shaolei Teng, Jayla Harvey, Christopher N. Cross

**Affiliations:** 1 Department of Biology, College of Arts and Sciences, Howard University, Washington, DC, 20059 USA; 2 Department of Anatomy, College of Medicine, Howard University, Washington, DC, 20059 USA

**Correction to: BMC Evol Biol**

**https://doi.org/10.1186/s12862-019-1516-y**

After publication of our article [1] we were notified that a few duplicate sentences were included on Figure 3 and Figure 4 legends.

Extra sentence removed from Figure 3 legend:

- The genomic region analyzed is given at the top of each plot (exons are labeled 1 through 4)

Extra sentence removed from Figure 4 legend:

- The distance from the core SNP (at zero) is displayed on the x-axis and the EHH values are shown on the y-axis

Also, in the sentence “In humans, MHC is a gene-dense region that spans ∼4 Mbs on the short arm of chromosome 6 and contains over 200 genes [113]”, “Mbs” was originally missing.

The original article has been corrected.
