# Supplementary material for: Multiple selective sweeps of ancient polymorphisms in and around LTα located in the MHC class III region on chromosome 6
Source: BMC Evol Biol. 2019 Dec 2;19:218. doi: 10.1186/s12862-019-1516-y (PMC6889576; doi:10.1186/s12862-019-1516-y)
Supplement: Supplementary file 1 — Additional file 1 : Figure S1. contains pairwise LD plots (spanning ~9.64 kbs) for African and non-African populations that were not included in Fig. 3 of the main manuscript. Figure S2. contains pairwise LD plots for loci located across a larger genomic region (spanning ~ 35.5 kbs) for each population. Figure S3. displays Manhattan plots of standardized |iHS| values in populations that were not included in Fig. 4 of the main manuscript. Figure S4. shows Manhattan plots of standardized |nSL| scores for all populations. Figure S5. presents the remaining EHH plots for populations that were not included in Fig. 4 of the main manuscript. [file 12862_2019_1516_MOESM1_ESM.pdf]

Supplementary Figure Legends

Figure S1. Pairwise linkage disequilibrium LD across a 9,637 base pair region in African and non-African populations. Pairwise plots of LD across ~10 kilobases, encompassing *LTα* and 5' region, were constructed using the `Haploview` program (40). The color of each square signifies the strength of the relationship between SNP alleles. For example, bright red squares indicate statistically significant LD between a given pair of SNP loci (D′=1; LOD > 2), while shades of pink/white squares indicate little evidence of LD. Purple squares indicate high LD but with little statistical support (low LOD) (40). The bold triangles indicate strong blocks of LD between SNP markers; specifically, the first and last markers in a given block are in strong LD with all intermediate markers. The genomic coordinate for each SNP marker listed along the top of the plot is given in Table S10. The plots labeled A-E are populations of African descent and plots labeled F-M are non-Africans. The gene structure of *LTα* and 5' region is at the top of each plot. The yellow boxes represent exons (exons are numbered 1 to 4), and the horizontal lines between the exons represent introns; the purple box immediately upstream of *LTα* is the 5' region.

Figure S2. Pairwise linkage disequilibrium (LD) across a 35,538 base pair region in African and non-African populations. Pairwise LD plots show the magnitude of association between SNP pairs in an ~35.5 kilobase region encompassing the *LTα* region and neighboring genes (namely, *NFKBIL1*, *TNFα*, and *LTβ)* for each population. The plots labeled A-G are individuals of African descent; H-L are South Asians; M-P are Europeans, while Q-U consist of East Asians. Magnitude of association is represented by white, purple and shades of red in the squares. Bright red squares indicate statistically significant LD between SNP pairs (D' = 100; logarithm of odds (LOD) > 2), whereas shades of pink/white cells signify little evidence of LD (D' < 100; LOD < 2) between loci; purple cells indicate high LD, but with little statistical support (low LOD) (40). The triangles encompassing SNPs indicated blocks of SNPs in strong LD between SNPs loci. The numbers along the top of the plots indicate marker numbers, which correspond to genomic coordinates given in Table S11. The yellow boxes represent exons and the horizontal lines between the exons represent introns; the purple box represents the upstream region of *LTα*. The

spaces between genes signify intergenic regions. Lastly, the 5' to 3' orientation of the genes are based on the genomic coordinates on chromosome 6 given in NCBI (129).

Figure S3. *i*HS statistic calculated for SNPs in African and non-African population. We computed the integrated haplotype score (*i*HS) (42) using the software `selscan` (43). To identify outlier values, the unstandardized scores for > 340,000 SNPs across the ~170 Mb region of chromosome 6 were normalized with the `norm` program implemented in the `selscan` package (43). SNPs with a standardized |iHS| > 2 represent the most extreme 5% of scores. This extreme threshold is represented by the horizontal dashed line. The red dots in plots represent outlier SNPs with |*i*HS| > 2 in and/or near *LTα*. The name of the population and the number of chromosomes analyzed are given at the top of each plot.

Figure S4. *n*SL statistic calculated for SNPs in select African and non-African population. We applied the *n*SL statistic which is a test for detecting both soft and hard sweeps (44) across the ~170 Mb region of chromosome 6. The output results were normalized following the same procedure used for *i*HS. SNPs with a standardized |*n*SL| > 2 represent the most extreme 5% of scores. This extreme threshold is represented by the horizontal dashed line. The red dots in plots represent outlier SNPs with |*n*SL| > 2 in and/or near *LTα*. The name of the population and the number of chromosomes analyzed are given at the top of each plot.

Figure S5. EHH performed in African and non-African populations. We applied the EHH statistic (45) to our data to identify signatures of recent positive selection based on long-range LD patterns on chromosomes containing core SNPs with extreme |*i*HS| and/or |*n*SL| scores. The SNP identifier for each core SNP are given at the top of the plots together with the name of the population and the number of chromosomes analyzed. The decay of LD for a given core SNP was measured by calculating EHH for the core SNP and the surrounding SNPs in the order of increasing distances on either side of the core SNP. EHH=0 means all extended haplotypes are different, while EHH=1 indicates that all extended haplotypes are the same. The plotted blue line represents the decay of homozygosity of chromosomes carrying the derived allele at the

core, while the plotted red line signifies the decay of homozygosity on chromosomes with the ancestral allele at the core SNP.

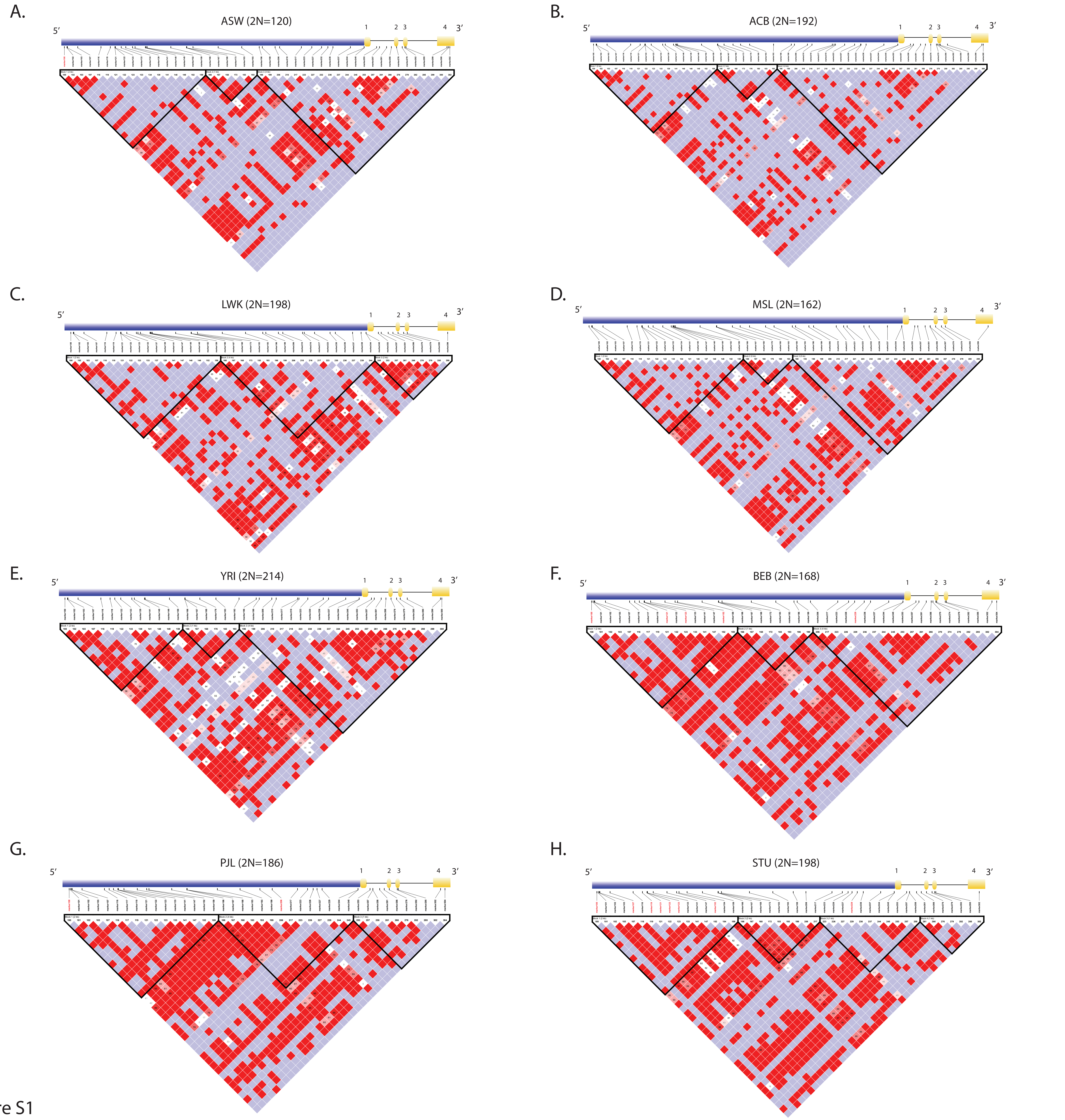
A.
ASW (2N=120)
B.
ACB (2N=192)
C.
LWK (2N=198)
D.
MSL (2N=162)
E.
YRI (2N=214)
F.
BEB (2N=168)
G.
PJL (2N=186)
H.
STU (2N=198)

Figure S1

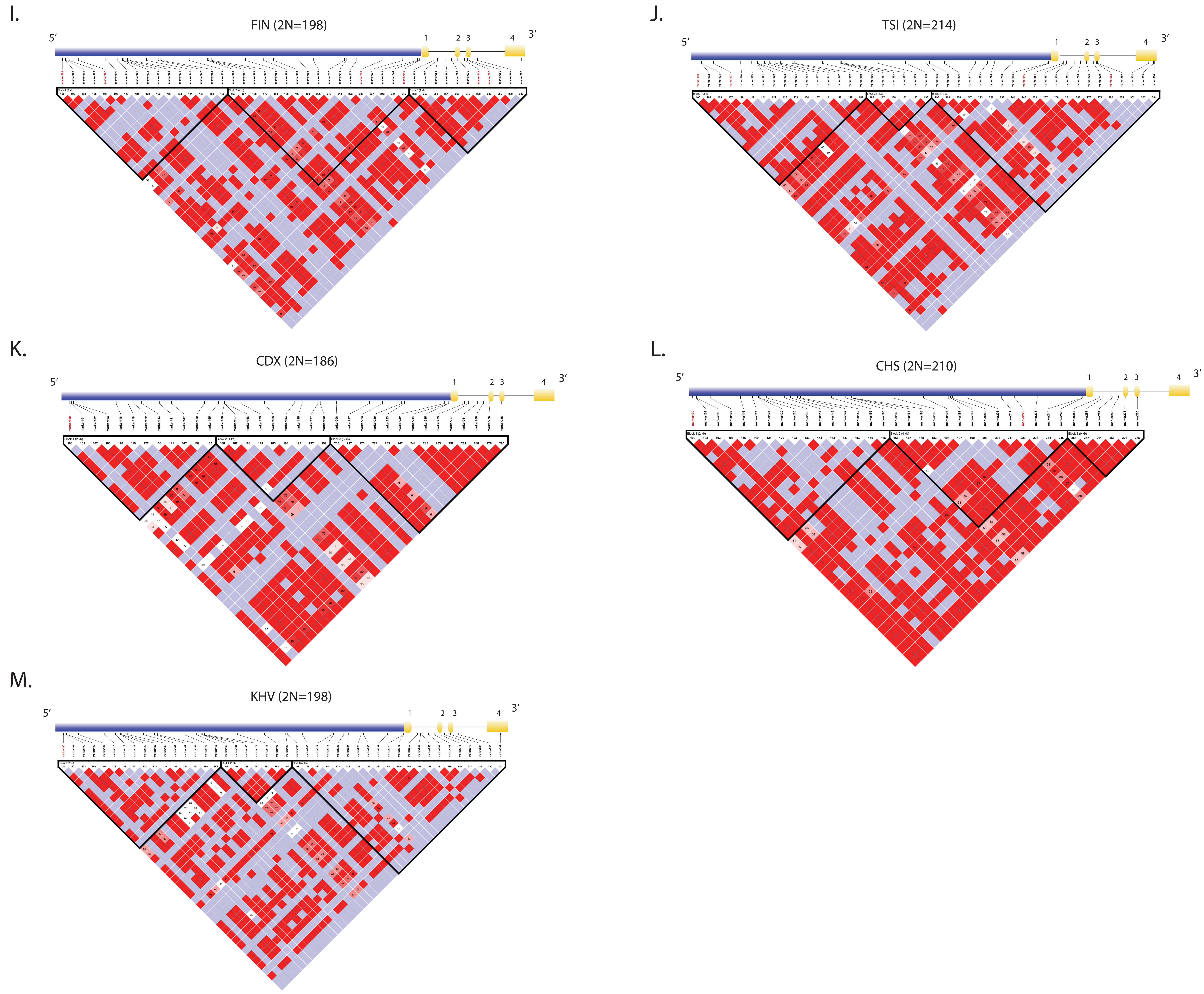
I.
FIN (2N=198)
5′
3′
1
2
3
4
J.
TSI (2N=214)
5′
3′
1
2
3
4
K.
CDX (2N=186)
5′
3′
1
2
3
4
L.
CHS (2N=210)
5′
3′
1
2
3
4
M.
KHV (2N=198)
5′
3′
1
2
3
4

Figure S1

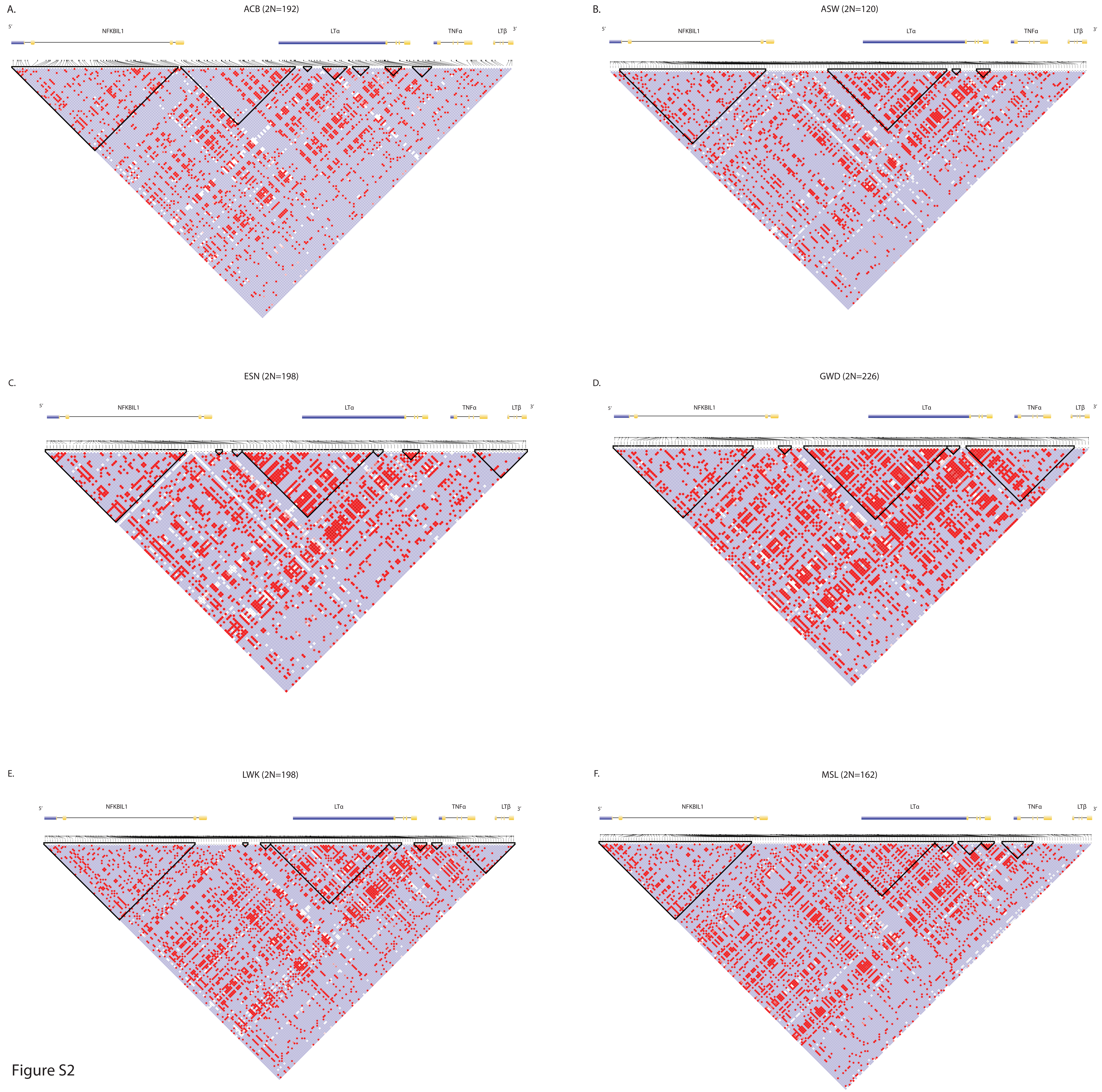
A.
ACB (2N=192)
5'
NFKBIL1
LTα
TNFα
LTβ
3'
B.
ASW (2N=120)
5'
NFKBIL1
LTα
TNFα
LTβ
3'
C.
ESN (2N=198)
5'
NFKBIL1
LTα
TNFα
LTβ
3'
D.
GWD (2N=226)
5'
NFKBIL1
LTα
TNFα
LTβ
3'
E.
LWK (2N=198)
5'
NFKBIL1
LTα
TNFα
LTβ
3'
F.
MSL (2N=162)
5'
NFKBIL1
LTα
TNFα
LTβ
3'

Figure S2

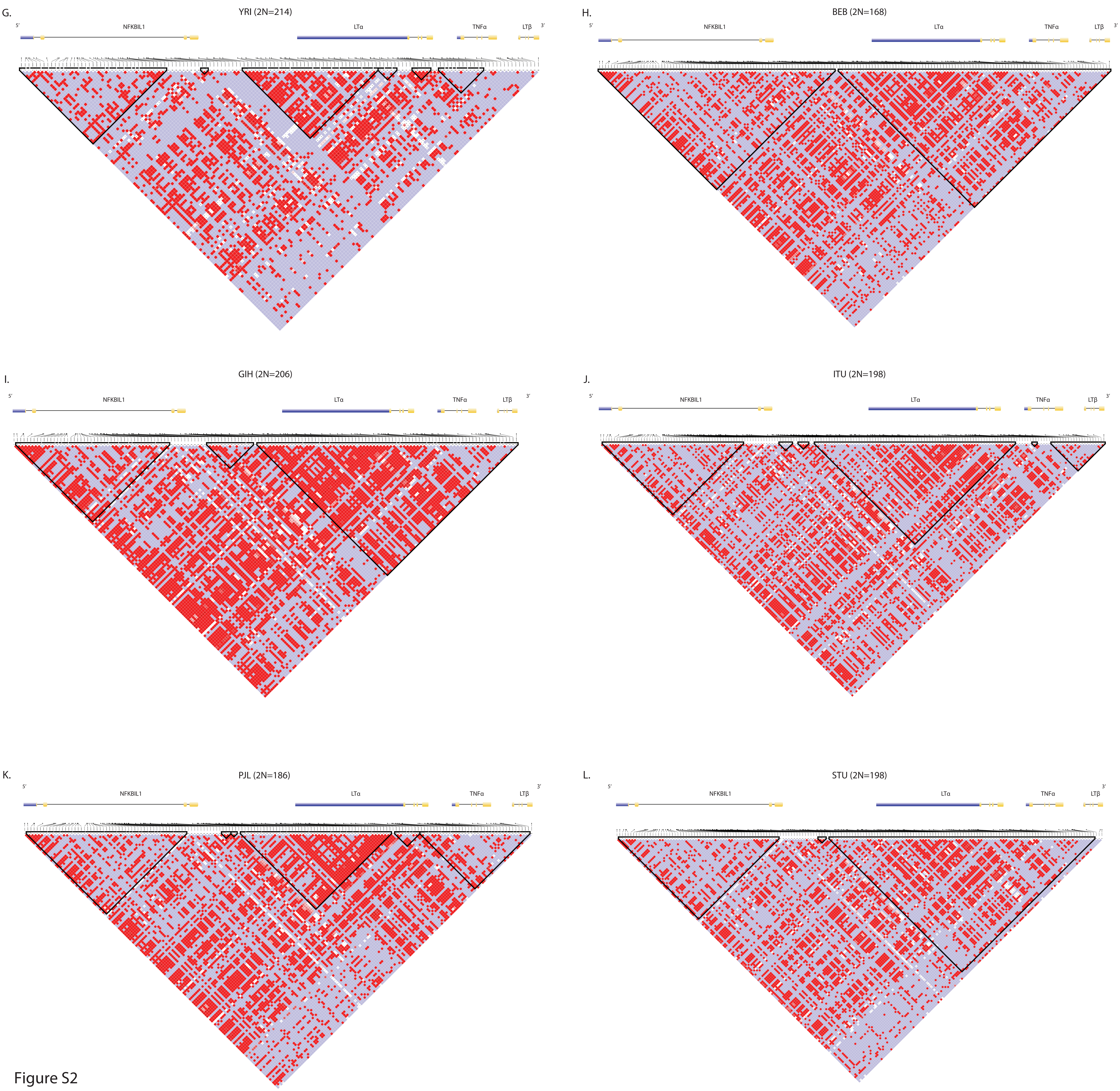
G.
YRI (2N=214)
5'
NFKBIL1
LTα
TNFα
LTβ
3'
H.
BEB (2N=168)
5'
NFKBIL1
LTα
TNFα
LTβ
3'
I.
GIH (2N=206)
5'
NFKBIL1
LTα
TNFα
LTβ
3'
J.
ITU (2N=198)
5'
NFKBIL1
LTα
TNFα
LTβ
3'
K.
PJL (2N=186)
5'
NFKBIL1
LTα
TNFα
LTβ
3'
L.
STU (2N=198)
5'
NFKBIL1
LTα
TNFα
LTβ
3'

Figure S2

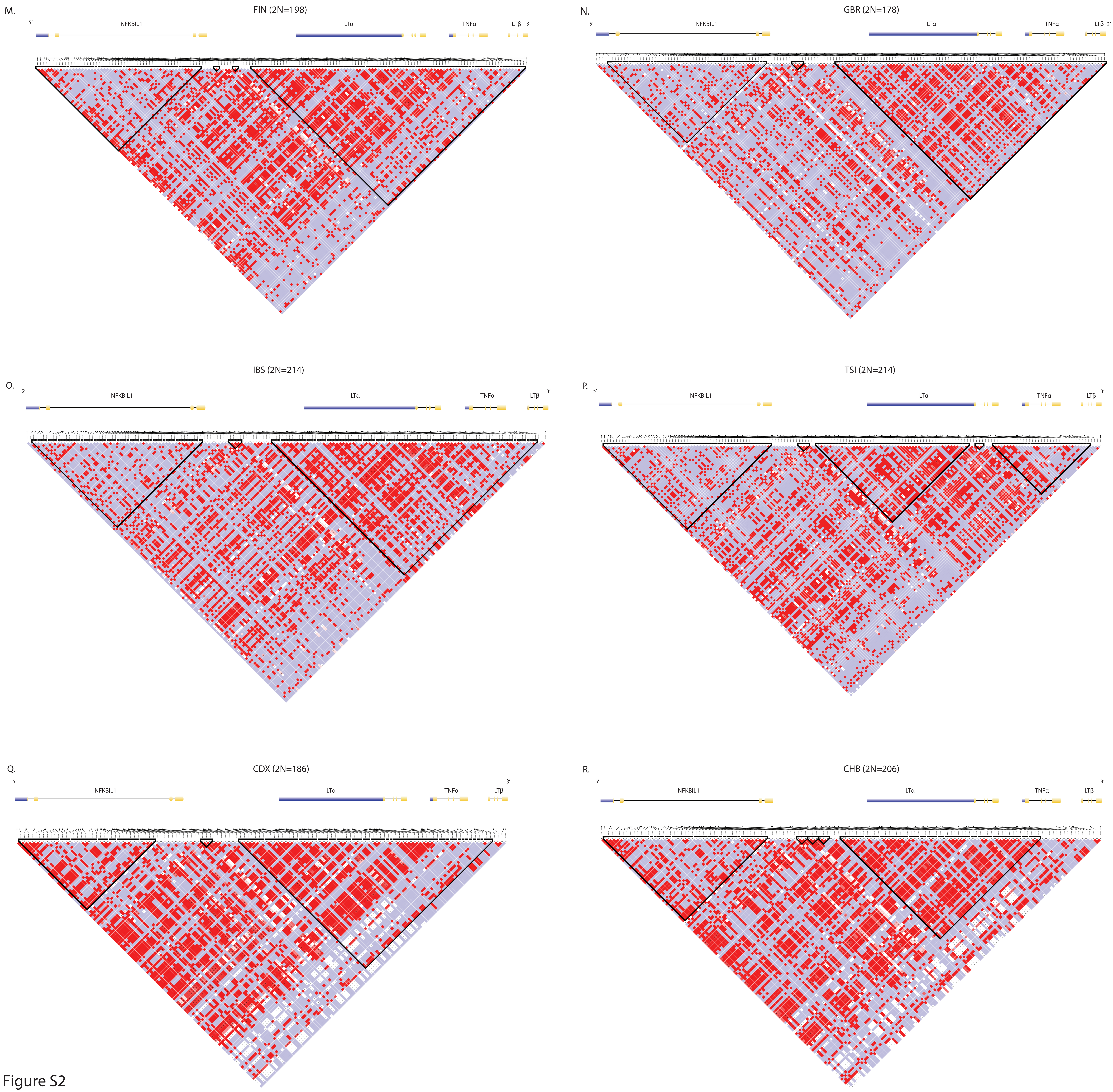
M.
FIN (2N=198)
5′
NFKBIL1
LTα
TNFα
LTβ
3′
N.
GBR (2N=178)
5′
NFKBIL1
LTα
TNFα
LTβ
3′
O.
IBS (2N=214)
5′
NFKBIL1
LTα
TNFα
LTβ
3′
P.
TSI (2N=214)
5′
NFKBIL1
LTα
TNFα
LTβ
3′
Q.
CDX (2N=186)
5′
NFKBIL1
LTα
TNFα
LTβ
3′
R.
CHB (2N=206)
5′
NFKBIL1
LTα
TNFα
LTβ
3′

Figure S2

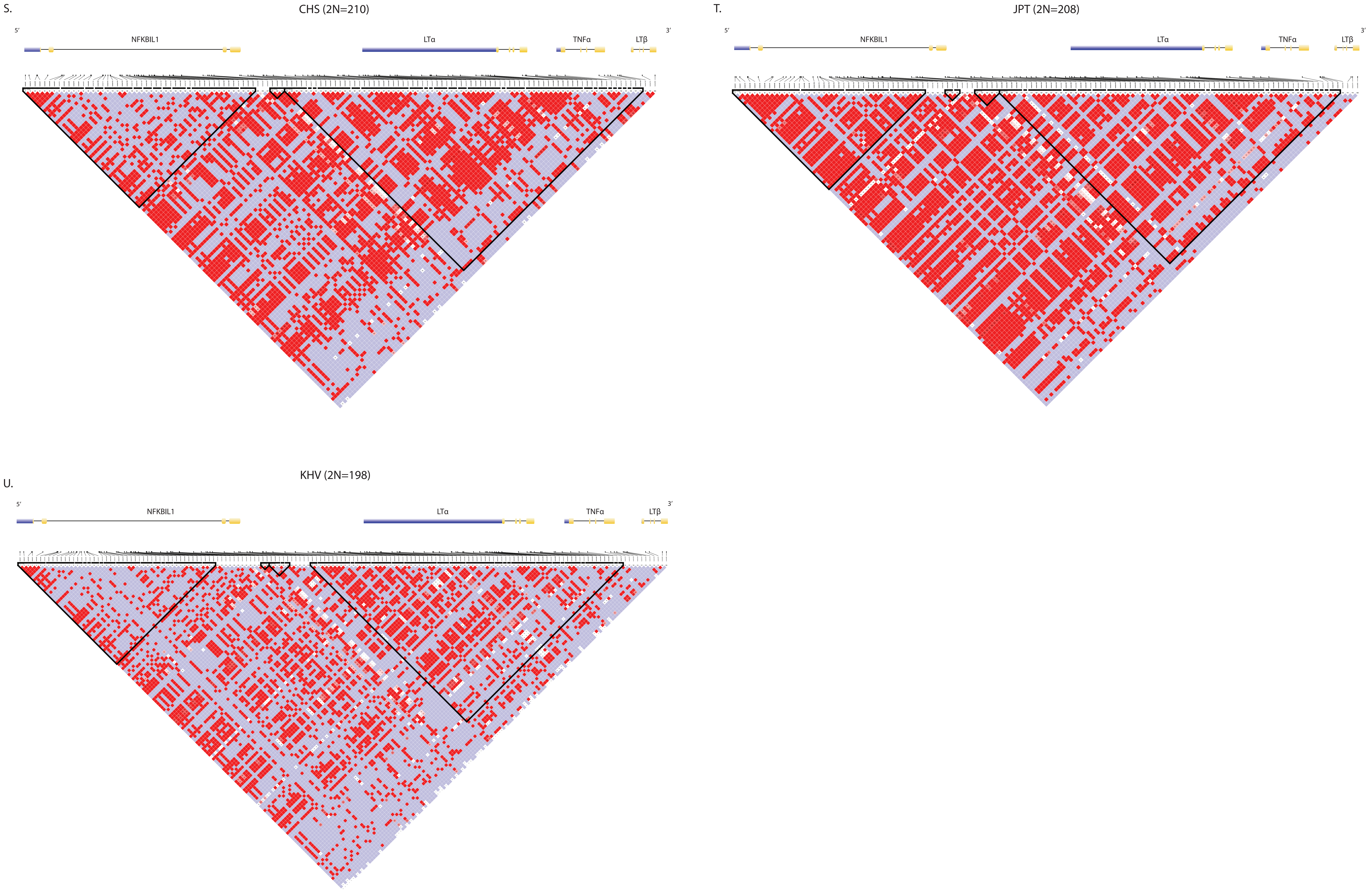
S.
CHS (2N=210)
5′
NFKBIL1
LTα
TNFα
LTβ
3′
T.
JPT (2N=208)
5′
NFKBIL1
LTα
TNFα
LTβ
3′
U.
KHV (2N=198)
5′
NFKBIL1
LTα
TNFα
LTβ
3′

Figure S2

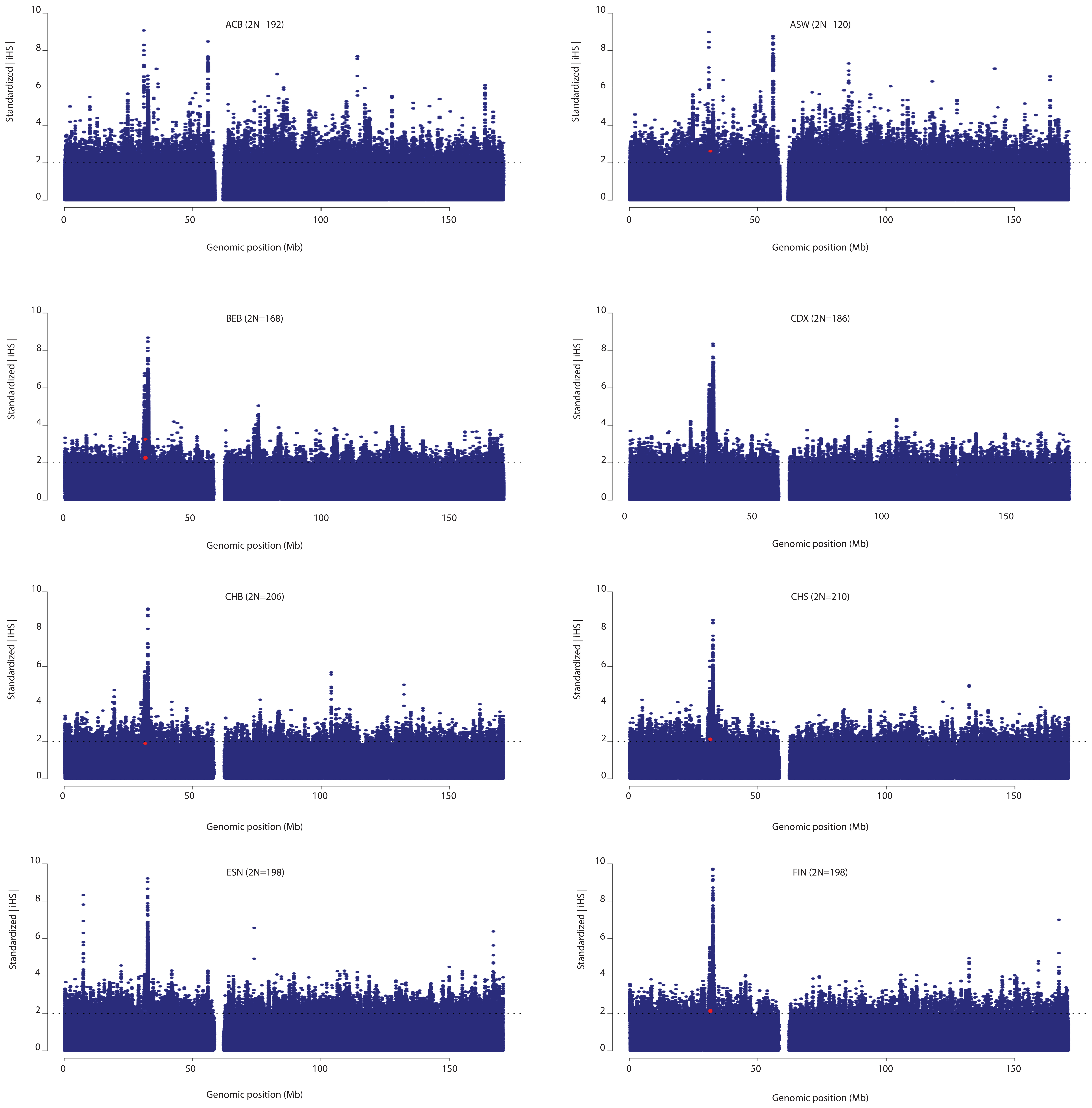
ACB (2N=192)
ASW (2N=120)
BEB (2N=168)
CDX (2N=186)
CHB (2N=206)
CHS (2N=210)
ESN (2N=198)
FIN (2N=198)
Standardized | iHS |
Genomic position (Mb)
0
2
4
6
8
10
0
50
100
150

Figure S3

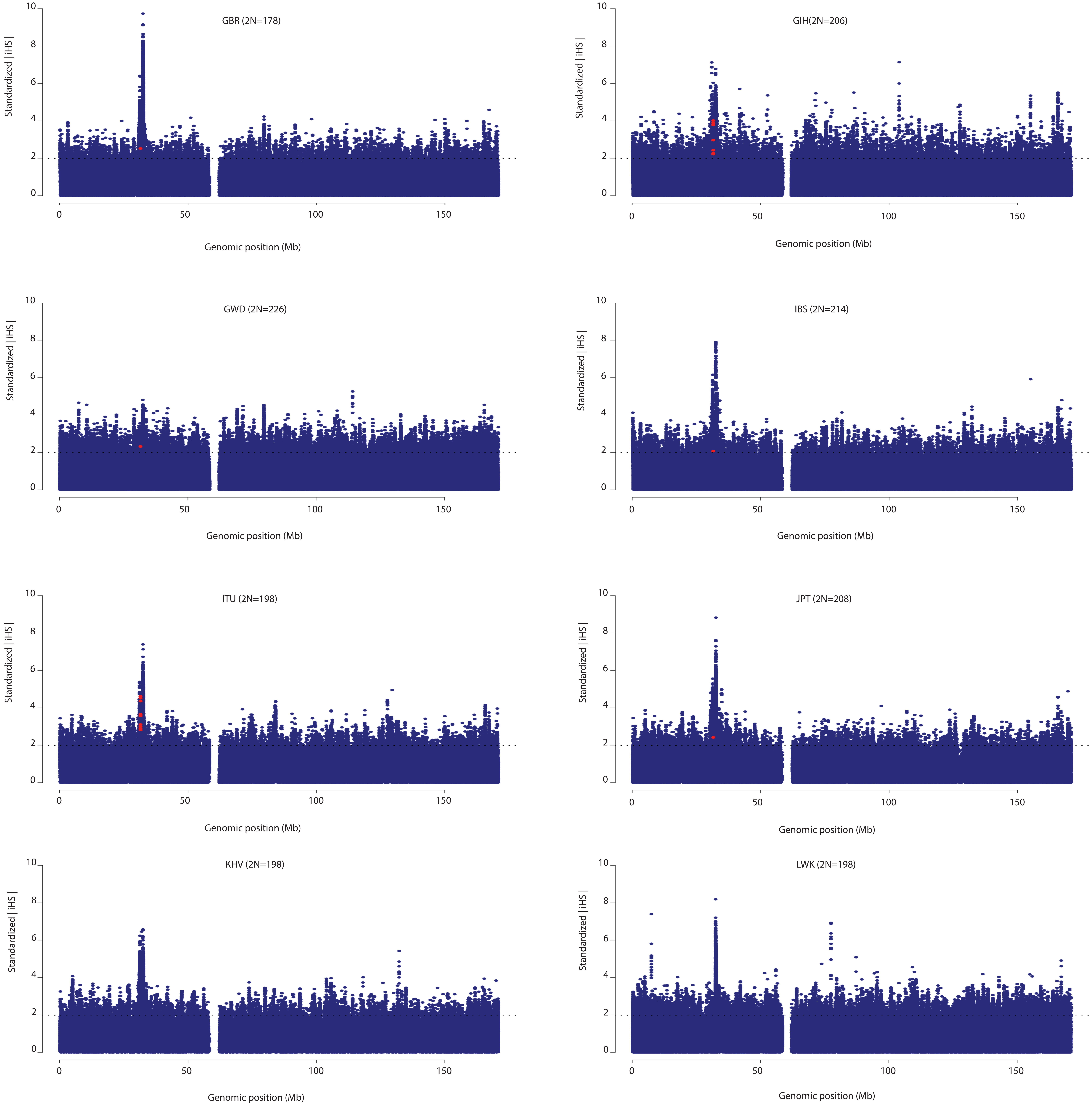
GBR (2N=178)
GIH(2N=206)
GWD (2N=226)
IBS (2N=214)
ITU (2N=198)
JPT (2N=208)
KHV (2N=198)
LWK (2N=198)
Standardized | iHS |
Genomic position (Mb)
0
2
4
6
8
10
50
100
150

Figure S3

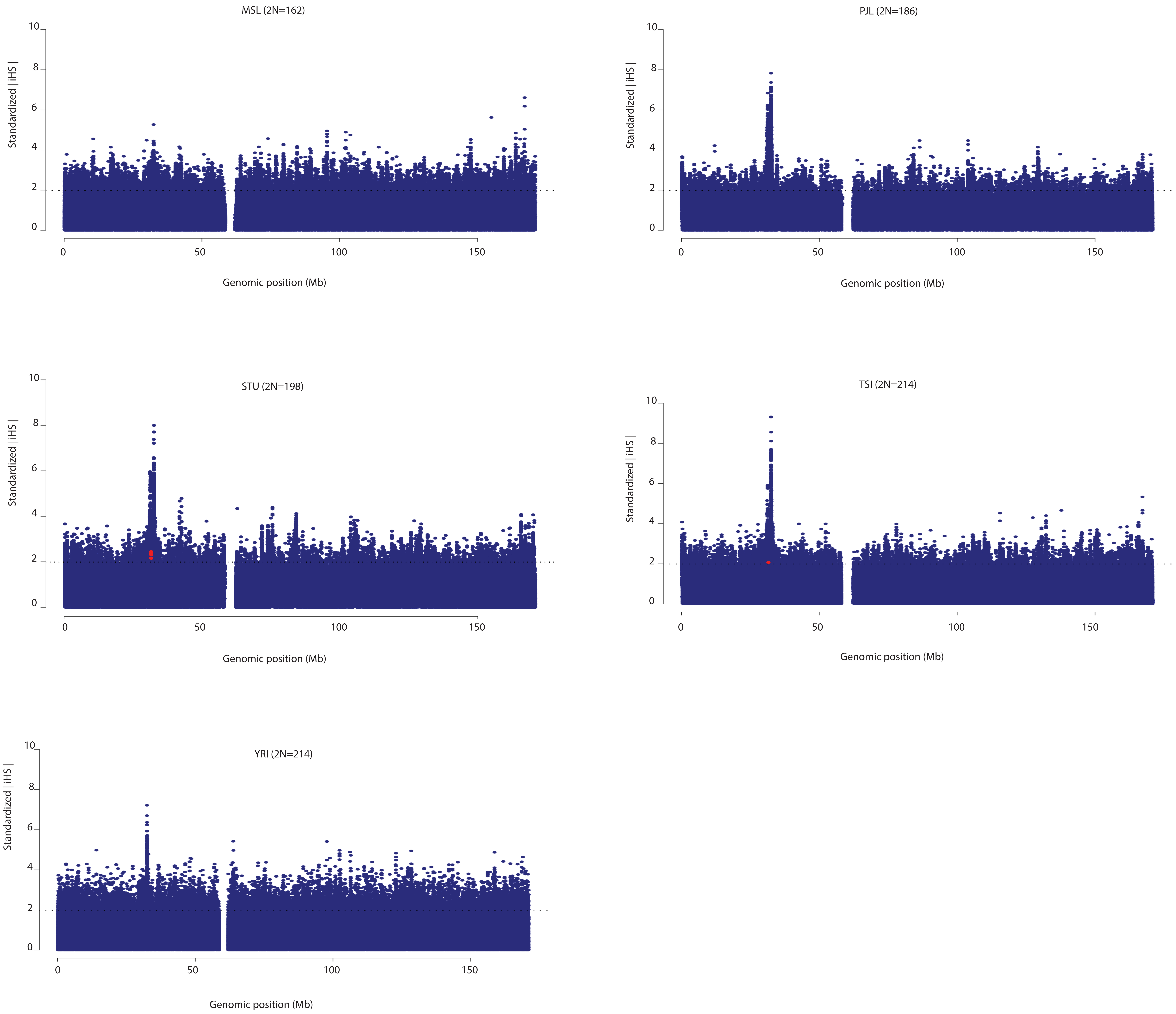

MSL (2N=162)
PJL (2N=186)
STU (2N=198)
TSI (2N=214)
YRI (2N=214)
Standardized | iHS |
Genomic position (Mb)
0
2
4
6
8
10
0
50
100
150

Figure S3

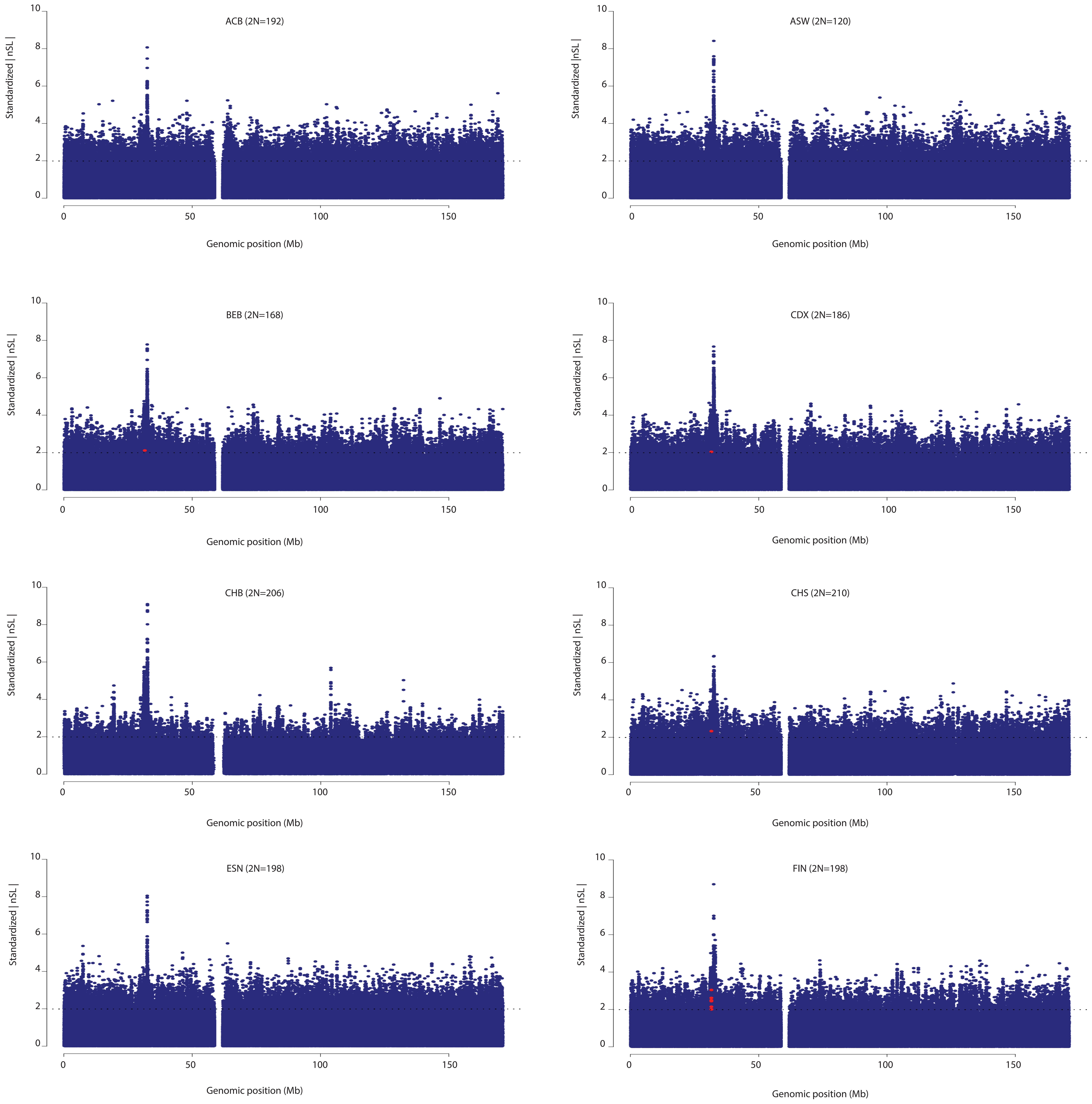
ACB (2N=192)
ASW (2N=120)
BEB (2N=168)
CDX (2N=186)
CHB (2N=206)
CHS (2N=210)
ESN (2N=198)
FIN (2N=198)
Standardized | nSL |
Genomic position (Mb)
0
2
4
6
8
10
50
100
150

Figure S4

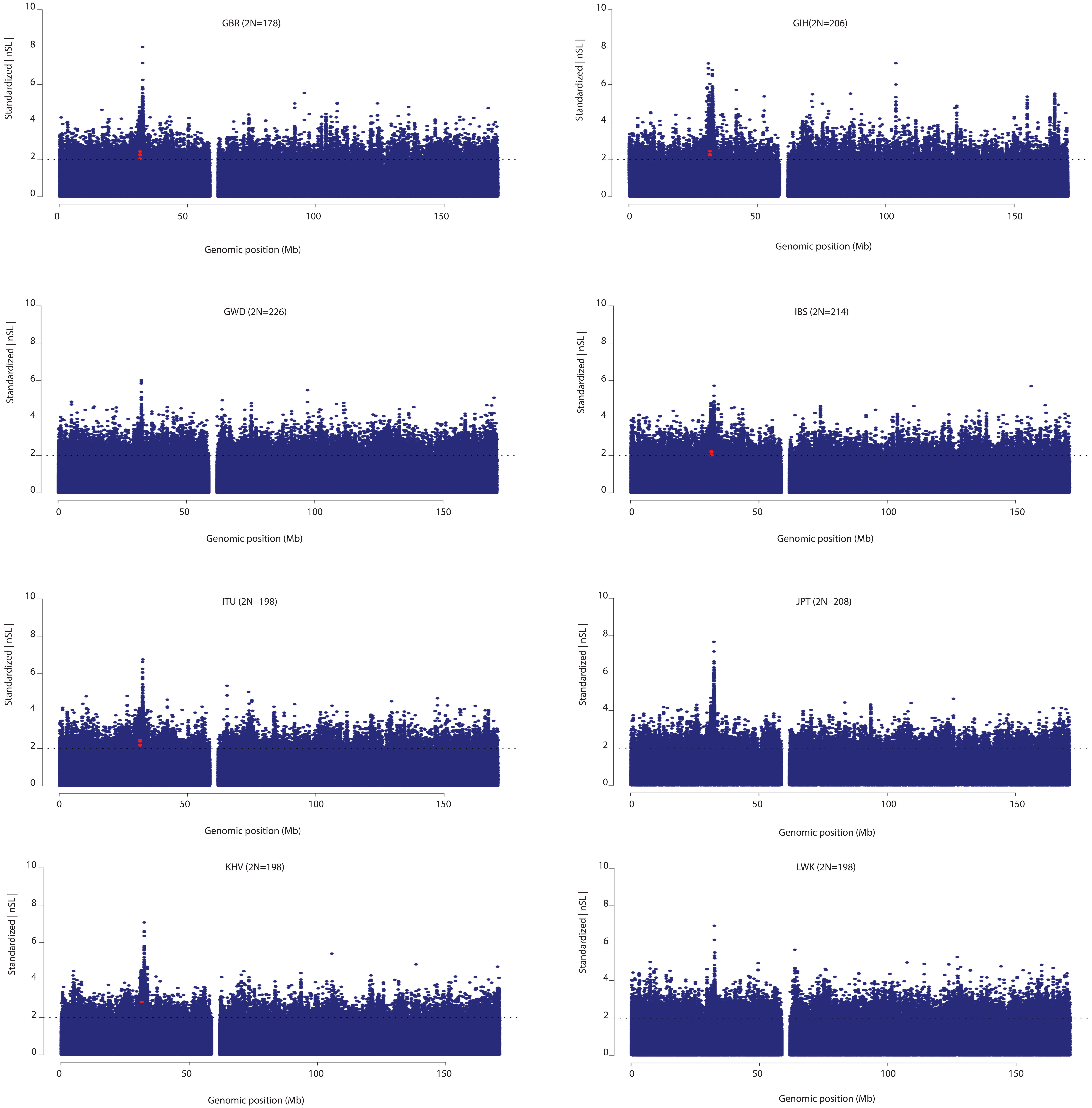

GBR (2N=178)
GIH(2N=206)
GWD (2N=226)
IBS (2N=214)
ITU (2N=198)
JPT (2N=208)
KHV (2N=198)
LWK (2N=198)
Standardized | nSL |
Genomic position (Mb)
0
2
4
6
8
10
50
100
150

Figure S4

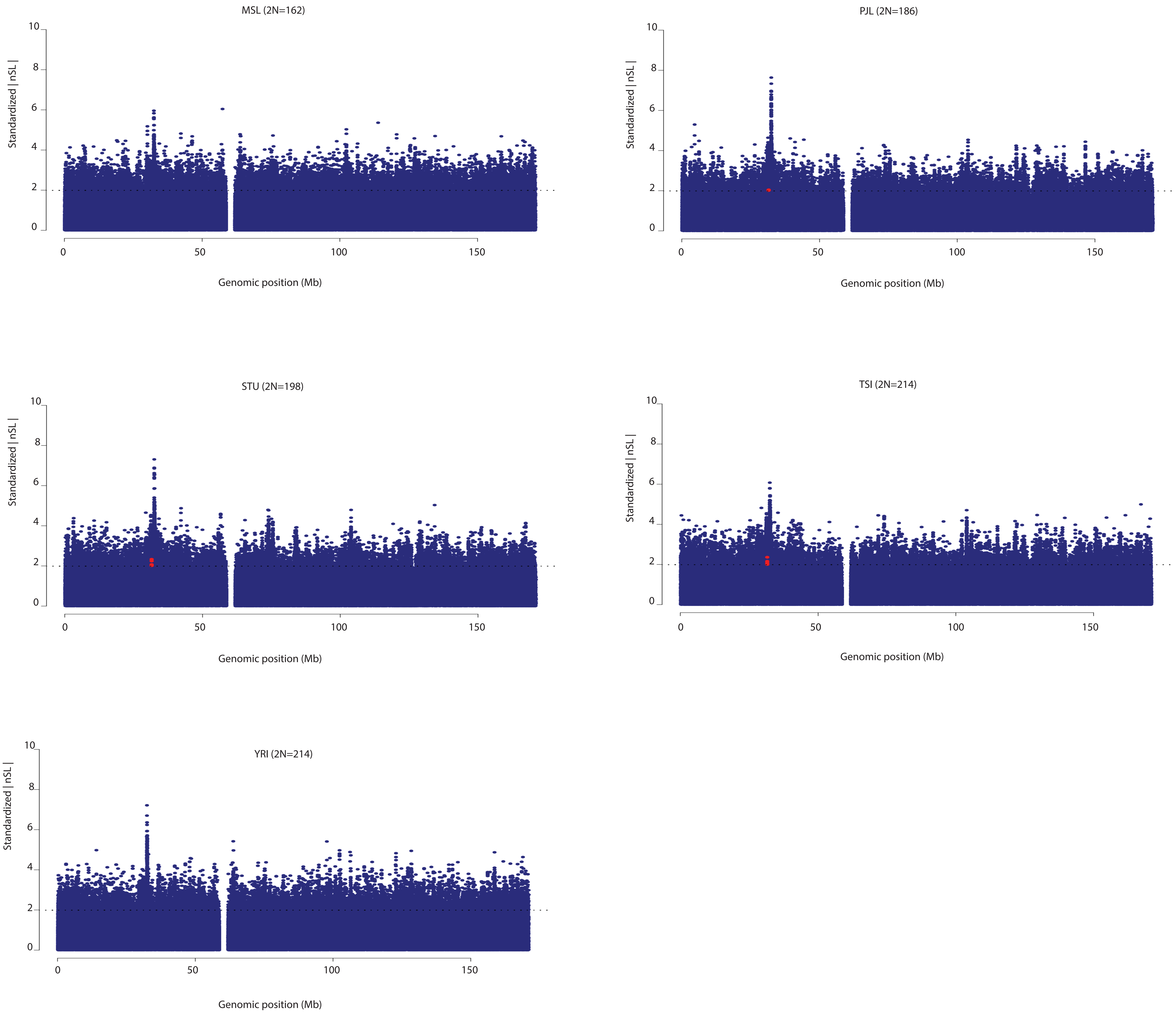
MSL (2N=162)
PJL (2N=186)
STU (2N=198)
TSI (2N=214)
YRI (2N=214)
Standardized | nSL |
Genomic position (Mb)
0
2
4
6
8
10
50
100
150

Figure S4

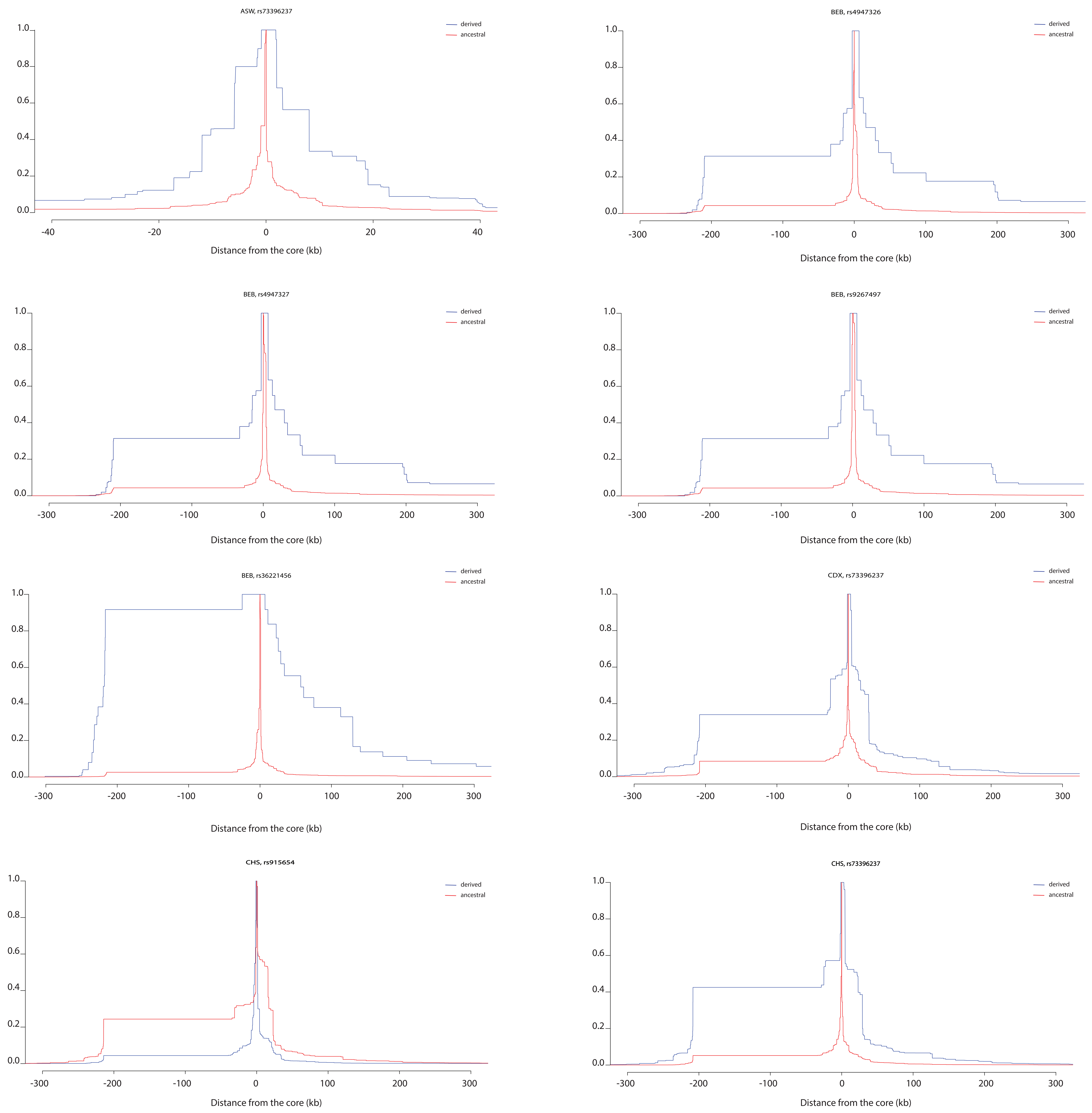
ASW, rs73396237
BEB, rs4947326
BEB, rs4947327
BEB, rs9267497
BEB, rs36221456
CDX, rs73396237
CHS, rs915654
CHS, rs73396237
derived
ancestral
1.0
0.8
0.6
0.4
0.2
0.0
-40
-20
0
20
40
-300
-200
-100
100
200
300
Distance from the core (kb)

Figure S5

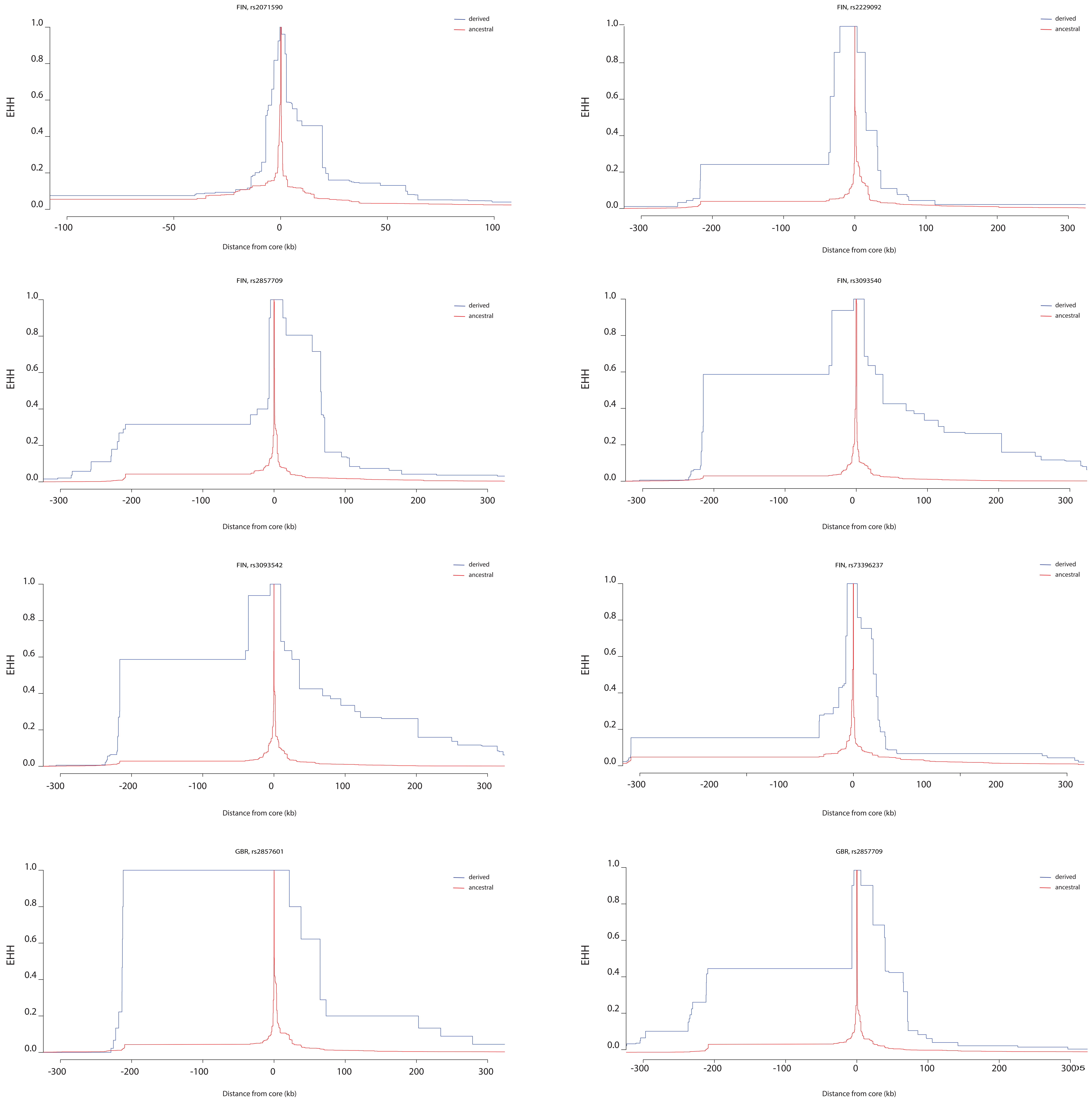
FIN, rs2071590
FIN, rs2229092
FIN, rs2857709
FIN, rs3093540
FIN, rs3093542
FIN, rs73396237
GBR, rs2857601
GBR, rs2857709
derived
ancestral
EHH
Distance from core (kb)
1.0
0.8
0.6
0.4
0.2
0.0
-100
-50
0
50
100
-300
-200
-100
0
100
200
300

Figure S5

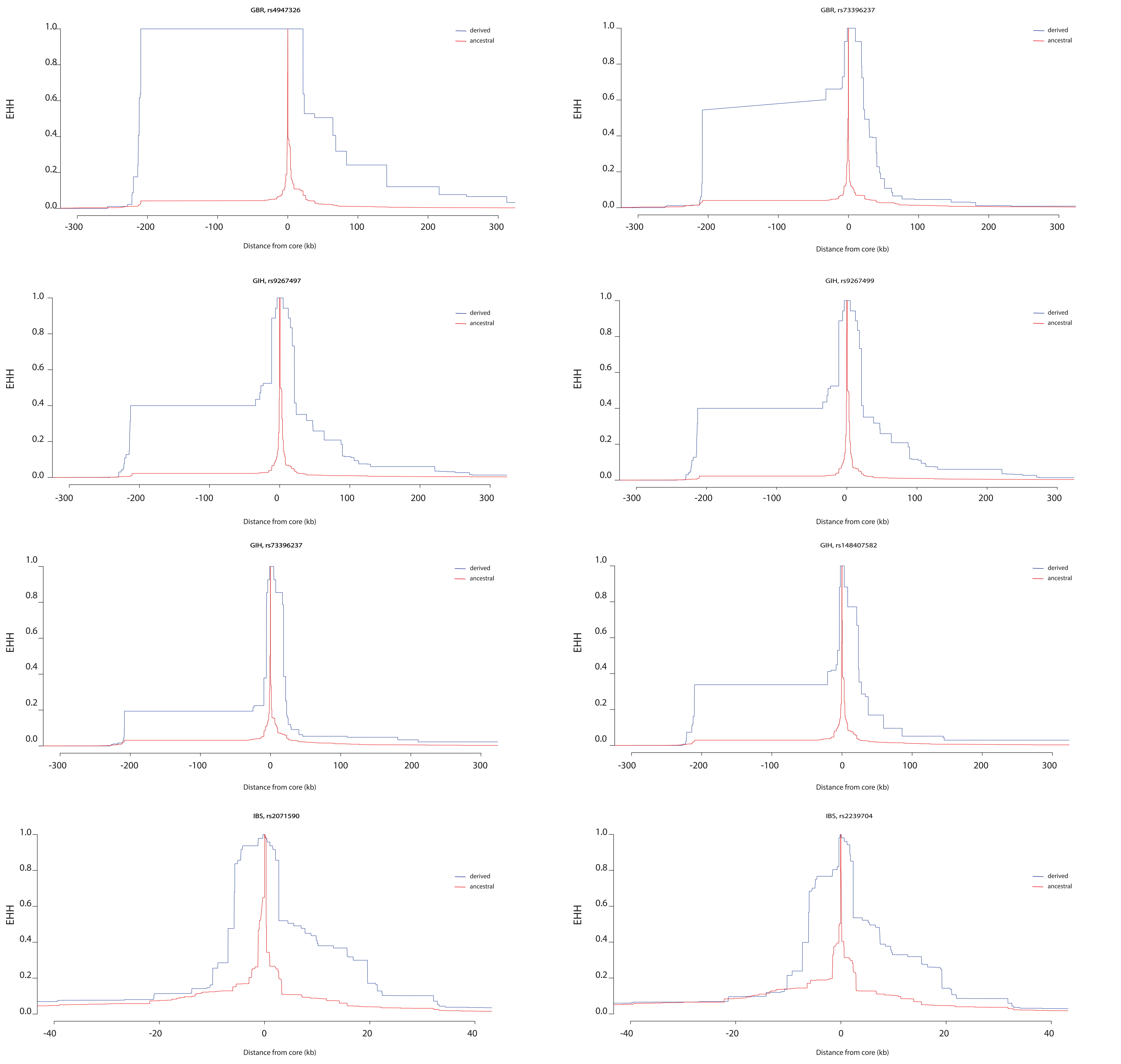
GBR, rs4947326
GBR, rs73396237
GIH, rs9267497
GIH, rs9267499
GIH, rs73396237
GIH, rs148407582
IBS, rs2071590
IBS, rs2239704
derived
ancestral
EHH
Distance from core (kb)
1.0
0.8
0.6
0.4
0.2
0.0
-300
-200
-100
0
100
200
300
-40
-20
20
40

Figure S5

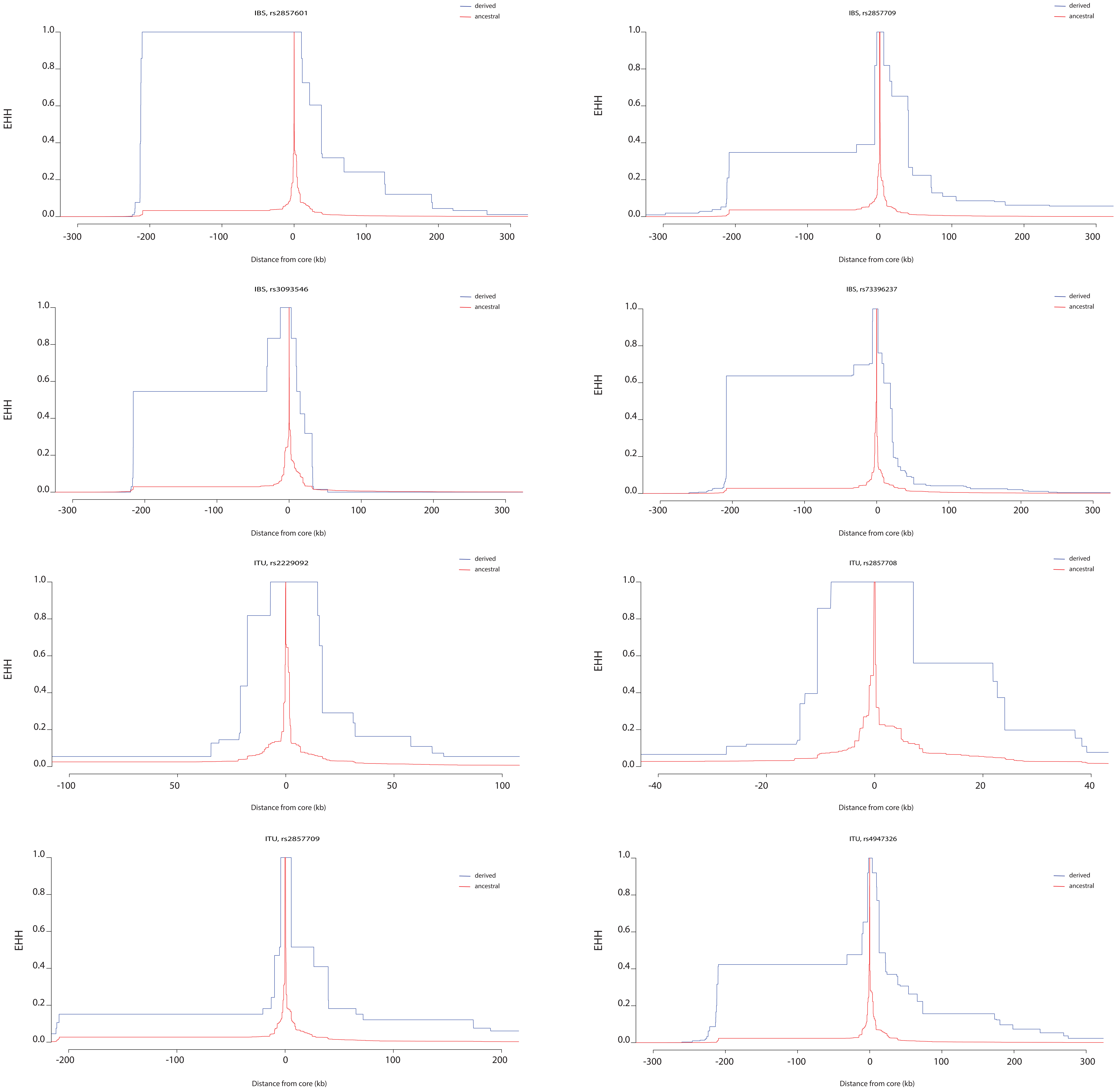

IBS, rs2857601
derived
ancestral
EHH
Distance from core (kb)
IBS, rs2857709
derived
ancestral
EHH
Distance from core (kb)
IBS, rs3093546
derived
ancestral
EHH
Distance from core (kb)
IBS, rs73396237
derived
ancestral
EHH
Distance from core (kb)
ITU, rs2229092
derived
ancestral
EHH
Distance from core (kb)
ITU, rs2857708
derived
ancestral
EHH
Distance from core (kb)
ITU, rs2857709
derived
ancestral
EHH
Distance from core (kb)
ITU, rs4947326
derived
ancestral
EHH
Distance from core (kb)

Figure S5

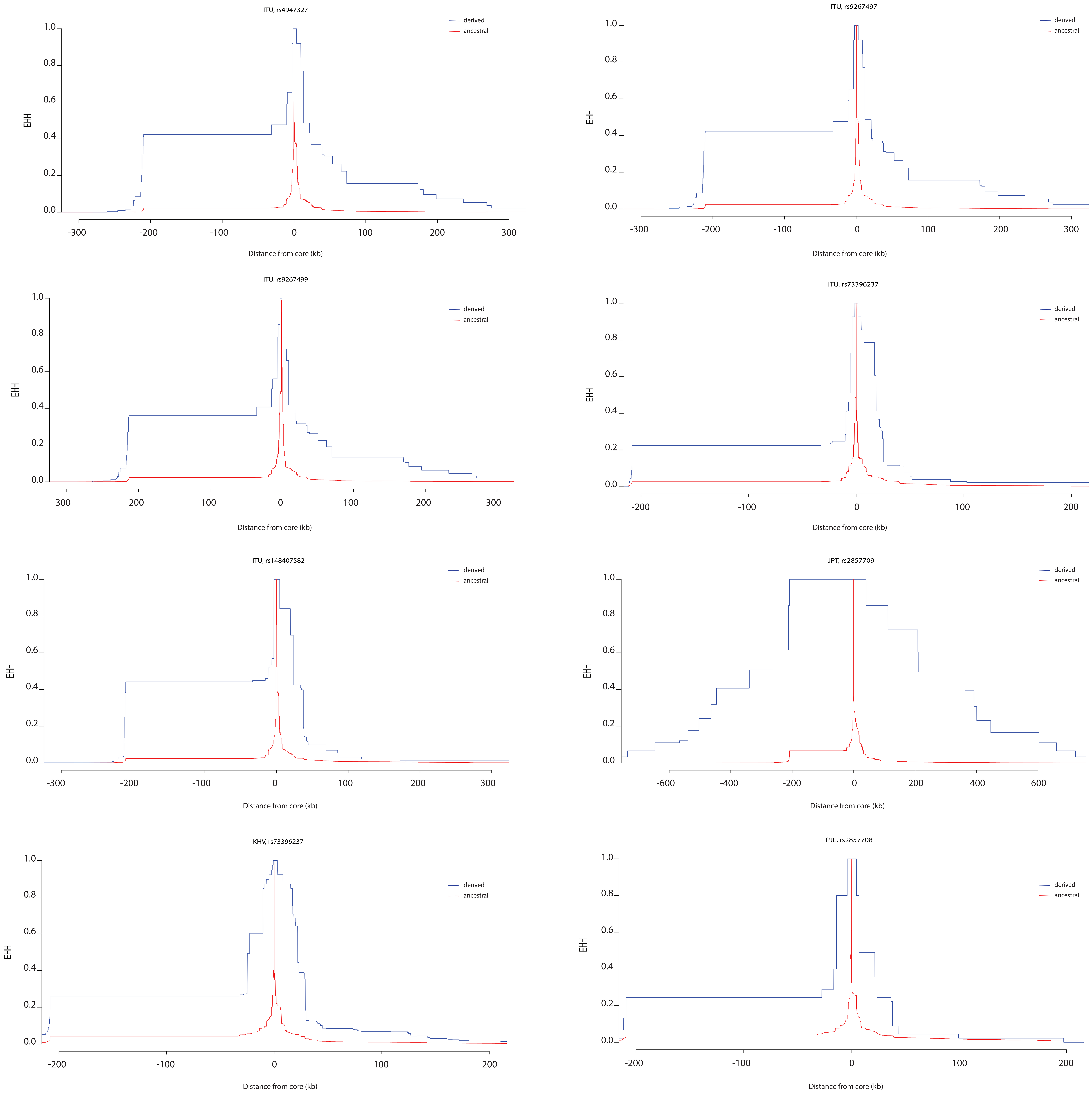
ITU, rs4947327
ITU, rs9267497
ITU, rs9267499
ITU, rs73396237
ITU, rs148407582
JPT, rs2857709
KHV, rs73396237
PJL, rs2857708
derived
ancestral
EHH
Distance from core (kb)

Figure S5

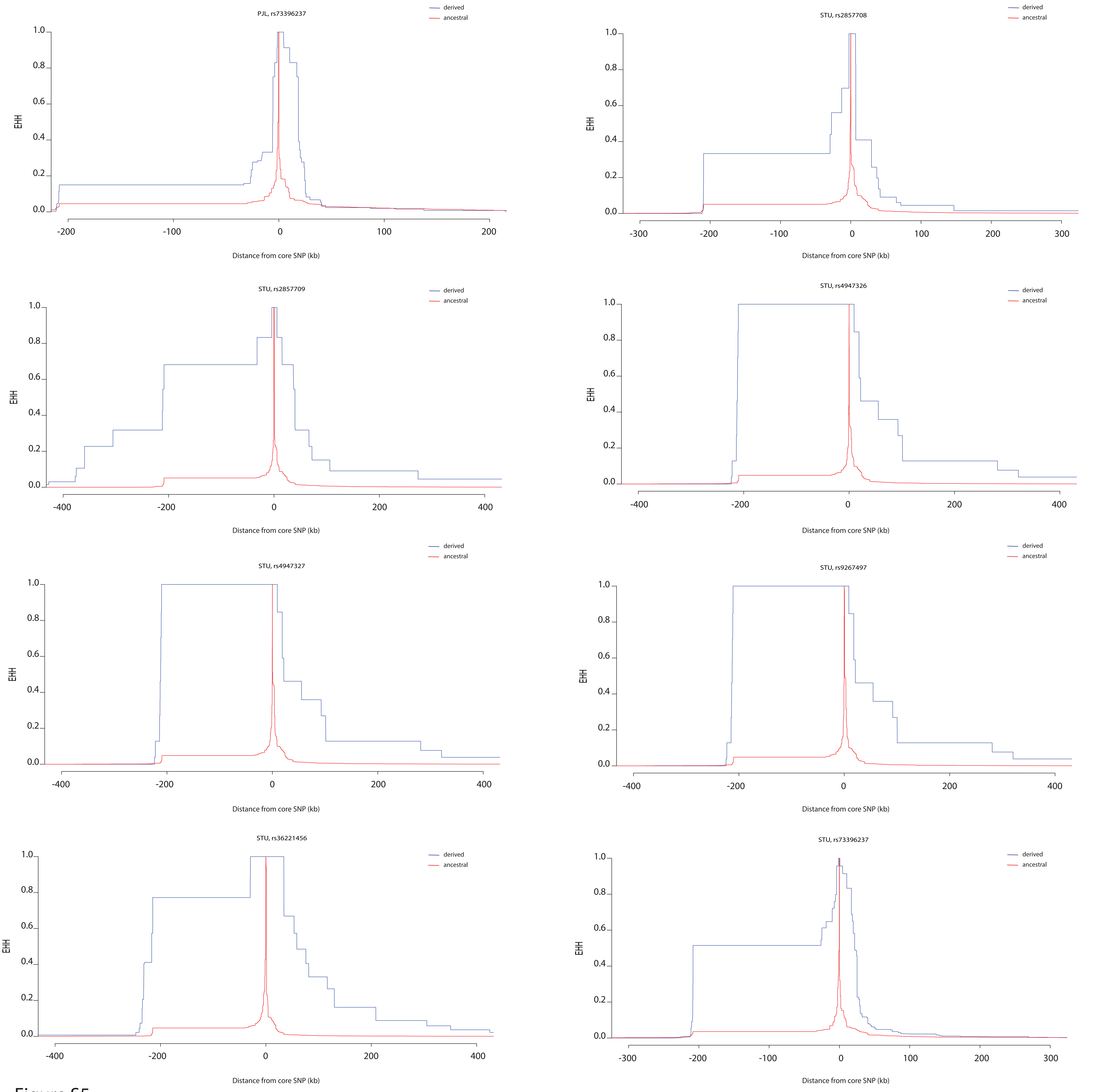
PJL, rs73396237
STU, rs2857708
STU, rs2857709
STU, rs4947326
STU, rs4947327
STU, rs9267497
STU, rs36221456
STU, rs73396237
derived
ancestral
EHH
Distance from core SNP (kb)

Figure S5

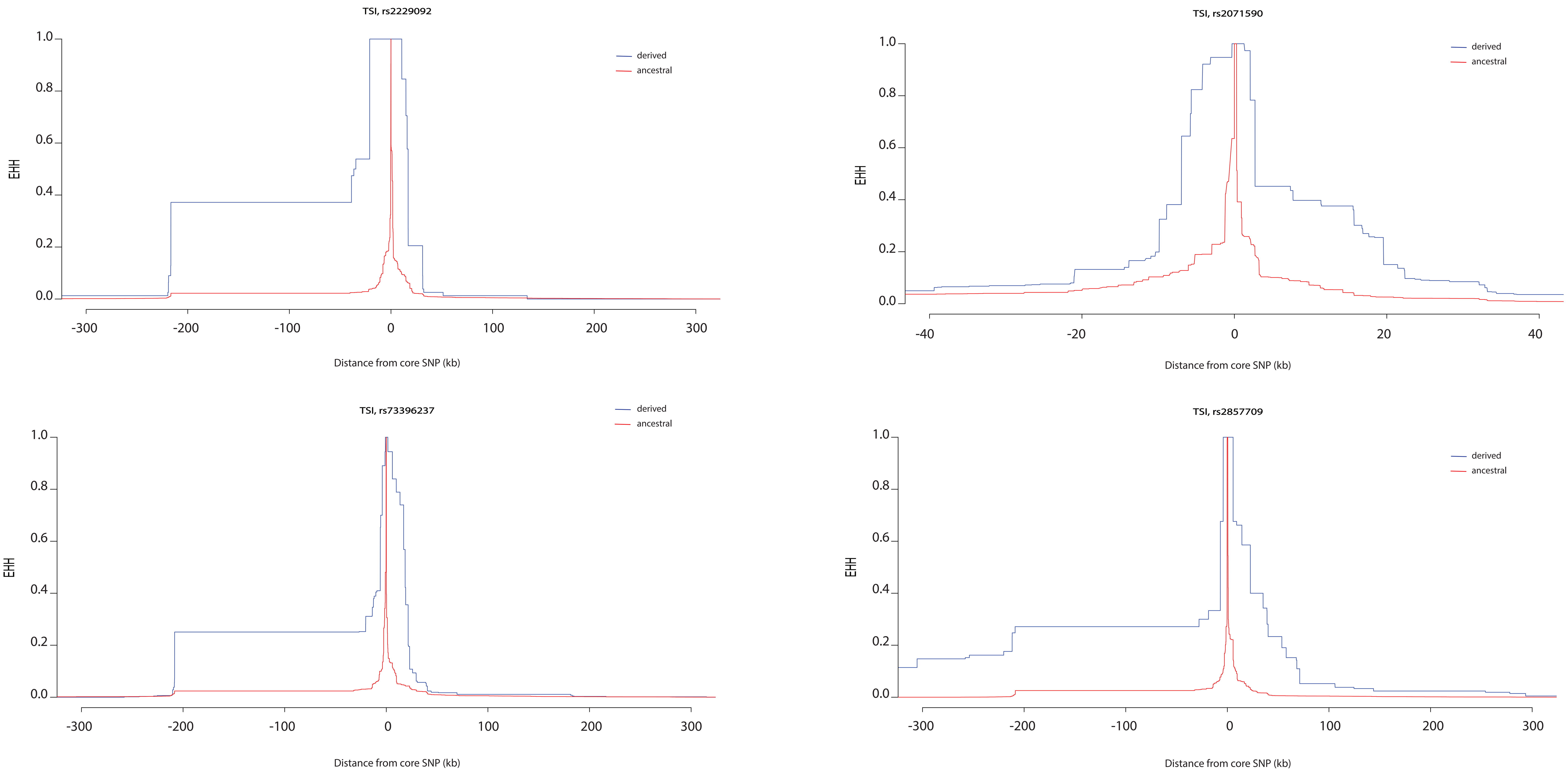
TSI, rs2229092
derived
ancestral
EHH
1.0
0.8
0.6
0.4
0.2
0.0
-300
-200
-100
0
100
200
300
Distance from core SNP (kb)
TSI, rs2071590
derived
ancestral
EHH
1.0
0.8
0.6
0.4
0.2
0.0
-40
-20
0
20
40
Distance from core SNP (kb)
TSI, rs73396237
derived
ancestral
EHH
1.0
0.8
0.6
0.4
0.2
0.0
-300
-200
-100
0
100
200
300
Distance from core SNP (kb)
TSI, rs2857709
derived
ancestral
EHH
1.0
0.8
0.6
0.4
0.2
0.0
-300
-200
-100
0
100
200
300
Distance from core SNP (kb)

Figure S5
